# Light quality characterization under climate screens and shade nets for controlled-environment agriculture

**DOI:** 10.1371/journal.pone.0199628

**Published:** 2018-06-25

**Authors:** Titta Kotilainen, T. Matthew Robson, Ricardo Hernández

**Affiliations:** 1 Organismal and Evolutionary Biology, Viikki Plant Science Centre (ViPS), Faculty of Biological and Environmental Sciences, University of Helsinki, Helinski, Finland; 2 Department of Horticultural Science, NC State University, Raleigh, NC, United States of America; United States Department of Agriculture, UNITED STATES

## Abstract

Climate screens are typically used inside glass greenhouses to improve control of humidity and temperature, and thus reduce energy expenditure. Shade nets are more appropriate to use, either with or without polyethylene cladding, at locations less-reliant on climate control, but where protection against hail, wind and excessive solar radiation might be needed. In addition, insect screens and nets can be employed to hinder insect pests and other invertebrates entering either type of production environment, and to keep invertebrates used in pest management contained inside. Screens and nets both transmit sunlight in a wavelength-specific manner, giving them the potential to affect plant morphology and physiology. Screens and nets of various colours and nominal shading factors have been described and studied; however, detailed measurements of their spectral characteristics are scarce. We measured solar spectral photon-irradiance and its attenuation by climate screens, shade nets, insect nets, greenhouse glass, and polyethylene covers. Our aim was to elucidate the effects of different patterns, colours, and shading factors, on light quality in production environments. Our measurements reveal that there are large differences both in the fraction of global irradiance attenuated and spectral ratios received under materials that are otherwise superficially similar in terms of their appearance and texture. We suggest that the type of spectral characterization that we performed is required to fully interpret the results of research examining plant responses to different types of screen and net. These data on spectral irradiance would benefit material manufacturers, researchers, growers, and horticultural consultants, enabling material selection to better match the solutions sought by growers and their desired outcomes regarding plant performance.

## Introduction

**Fig 1:**
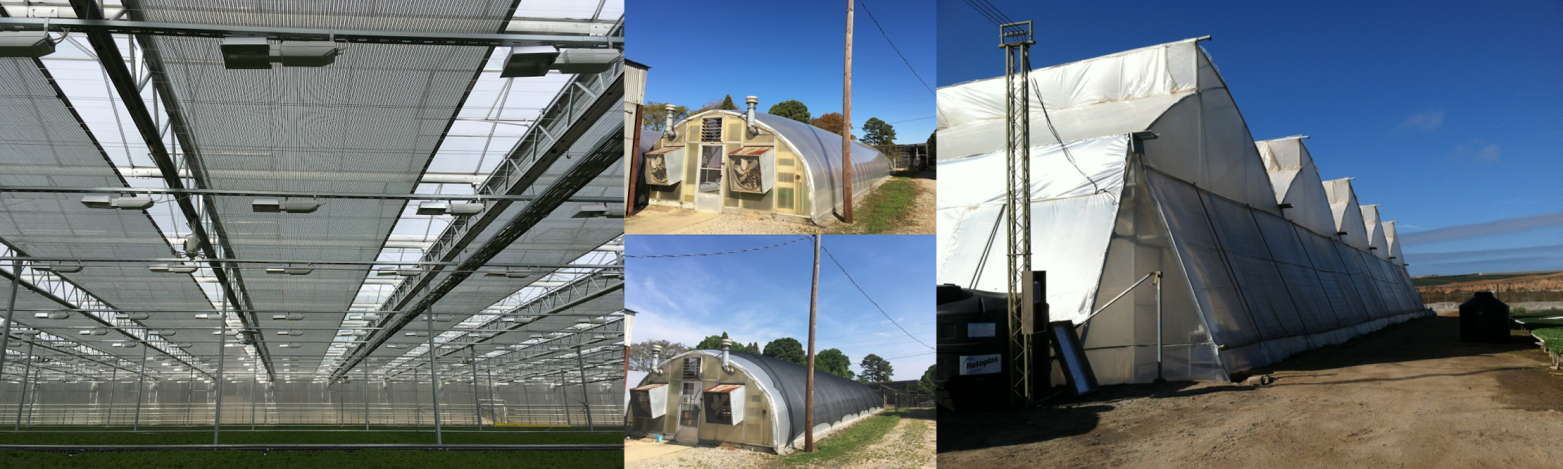
Photos showing examples of the use of climate screens and shade nets. Left: a glass greenhouse equipped with climate screens; middle up: a polytunnel during the cooler season; middle down: a polytunnel during the warm season equipped with a shade net; right: a shade house.

Climate screens and shade nets have two main uses in controlled-environment horticulture. (1) Screens can be employed inside a greenhouse to improve climate control and save on the energy expended in heating and cooling. They are deployed automatically in greenhouses when certain environmental thresholds are surpassed (Fig 1). (2) Shade nets can be used to cover lightweight trellis structures (shade houses), and are sometimes combined with plastic covers (Fig 1). These types of structures are less expensive than glass or plastic greenhouses, and are used in regions of the world and for applications where protection and some shading are needed more than climate control. Nets can be used as vertical windbreaks and horizontally on structures without sidewalls. The shade nets used in these types of application can be made of several different materials, patterns, and colours, and their main purpose is to protect plants from abiotic and biotic damage such as hail, wind, birds, and excessive solar radiation. A specific sub-group of screens and nets are those designed to be used for insect control [1–3]. The effects of climate screens and shade nets on the microclimate; air flow; ventilation rate; temperature; humidity; the transmittance of photosynthetically active radiation (PAR) and near-infrared radiation (NIR); as well as, crop water use efficiency and productivity (yield and quality) are reviewed by Tanny [1] and Ahemd et al. [4].

The spectral composition of light received by plants (spectral quality) can affect their morphology, physiology, and growth rate. In addition to reducing the total spectral irradiance (radiation incident on a flat surface per unit area), climate screens and shade nets can attenuate sunlight in a wavelength-specific manner. This means that when selecting a screen/net for commercial use, it would be wise to consider whether its selective absorption is likely to produce plants with desirable characteristics for a given purpose. For example, by selecting a shade net that produces a spectrum high in blue light, that reduces stem extension [5], a grower might obtain a more attractive and compact plant without recourse to labour-intensive and time-consuming pruning practices or the use of chemical growth regulators [6]. Likewise, produce quality can be improved by the choice of an appropriate shade net. A study of different-coloured nets found that, in comparison with yellow nets, red and pearl nets improved tomato fruit mass, firmness, and bioactive components (ascorbic acid, lycopene, beta-carotene and total phenols) in two out of three cultivars tested, but the effects of the different nets on spectral quality were not recorded [7].

According to Kitta et al. [8], most studies of shade nets consider only the reduction they cause in total irradiance and not their effect on spectral composition. Both shade net and climate screen manufacturers calculate the shading factor (SF, %) for this purpose, describing the relative proportion of radiation that is absorbed and reflected either in the visible range (380–760 nm) or photosynthetically active radiation PAR range (400–700 nm). Some climate screen manufacturers also report separate shading factors for direct and diffuse radiation. These values are affected by the fabric colour, mesh size, and texture of the screen/net [3].

The effects of different shade nets on the spectral quality of sunlight in greenhouses and polytunnels have been assessed in some studies. Kittas et al. [9] compared the radiation inside a glass greenhouse when it was equipped either with an external black shade net, an internal aluminized thermal screen, or a white-painted roof. Equivalent measurements of spectral irradiance were also made in a polyethylene greenhouse, greenhouse made with fibreglass panels, and a polyethylene tunnel. Small changes in the PAR to NIR photon ratio indicated that the glass and white-painted roof enriched PAR relative to NIR inside the greenhouse, whereas the two plastic covering materials, polyethylene and fibreglass, tended to enrich the NIR relative to PAR. Similarly, Arthurs et al. [6] monitored light quality, temperature, relative humidity and wind resistance in polytunnels equipped with red, blue, pearl and black nets (all ChromatiNet®, Polysack Plastic Industries, D.N. Negev, Israel) over a one year period. Pearl nets were most effective at reducing the transmittance of both UVB radiation (280–315 nm) and UVA radiation (315–400 nm), while red nets reduced transmittance of UV radiation the least. Black nets reduced the transmittance of PAR the most (by 55–60%), and red nets the least (by 41–51%), while blue and pearl nets were intermediate. Pearl nets transmitted more sunlight than the black nets in the region of the spectrum above 400 nm but did not alter the spectral composition of light in the visible range. Blue nets had distinctive peaks in transmittance in the blue waveband (defined as 450–495 nm) and far-red wavelengths beyond 750 nm. Red nets allowed approximately 50% transmittance around 400 nm wavelength, but produced over 70% transmittance at wavelengths beyond 590 nm. Pearl, black and red nets gave R:FR ratios (defined as 600–700 nm/700-800 nm) similar to ambient (R:FR ratio approaches 1.0), whereas blue nets lowered the R:FR ratio to around 0.8. In all, the general tendency is that black nets do not greatly alter spectral quality under them, pearl nets can reduce the transmittance of UV-radiation, and blue and red nets alter spectral quality more in the PAR/visible range.

Several studies describing the effects of coloured nets on crop growth and yield have provided some information on light quality [7,9–11]. These types of nets are also used in pest and pathogen management [12–14]. Unfortunately, all reported irradiance measurements from studies of coloured nets (except Arthurs et al. [6]), lacked wavelength-specific data on UV irradiance. Standard greenhouse glass does not transmit UVB radiation; however, in climate-controlled greenhouses where climate screens are used, greenhouse roof vents open for ventilation allowing some solar UVB radiation to enter. This means that the relative transmittance of UVB radiation and the UVB:PAR ratio of climate screens are relevant in determining the UVB radiation in the interior environment, since even a relatively low fluence of UVB radiation can elicit plant responses [15,16]. In addition, the response of plants to changes in the UVB:PAR ratio has been widely studied in the context of stratospheric ozone depletion. One finding of this line of research is that plants grown under higher UVB:PAR ratio (lower PAR) tend to be more sensitive to UVB radiation than those grown under a realistic UVB:PAR ratio [17,18]. It has been suggested that the role of UVB radiation is multifaceted, i.e. when the UVB:PAR ratio is low UVB radiation acts as a regulator of photomorphogenesis, but when the UVB:PAR ratio is high UVB radiation can be a stressor [19]. If the vents in the roof open to cool the greenhouse at midday when UVB irradiance is highest, there is a large and acute increase in UVB radiation and the UVB:PAR ratio that is potentially very stressful to the plant. UVA radiation can cause distinct plant responses to those elicited by UVB radiation and blue light [20], because the action spectra of different plant photoreceptors pass through the UVB (UVR8) and UVA (cryptochromes, phototropins, and zeitlupe proteins) wavebands. The distinct functions of these photoreceptors may be responsible for some of the contradictory reports of UV-mediated plant responses.

Furthermore, wavebands and their ratios are often reported in an inconsistent manner, i.e. using different definitions for spectral wavebands. This makes comparisons between studies difficult [7,10,11]. In addition, all the published measurements of spectral irradiance are from shade nets, and no detailed spectral quality measurements of climate screens are available.

In all, only a few studies have attempted to characterize and compare detailed light quality conditions under the available range of greenhouse materials and shade nets. Yet the consequences of effects on the light environment are potentially far-reaching, as a large estimated area, some 3,414,353 ha worldwide in 2017 [21], is covered by greenhouses, polytunnels and shade houses. The economic importance of this sector is highlighted by a survey of commercial plant production across fifteen US states by the USDA [22], which found 6533 ha of covered production environments in 2015 with a turnover higher than $100,000 per year. Of this 6533 ha, 7.5% was in floricultural crop production under glass greenhouses, 8.2% was under fiberglass or other rigid material, 37.2% was under plastic, and 47.1% was under shade nets or other temporary covers [22].

Our aims were to describe in detail the spectral characteristics of; (1) climate screens commonly used in year-round production greenhouses of various constructions requiring precise climate control; (2) nets used for shading and insect control by themselves (i.e. shade houses) or in polytunnels, and (3) to place our measurements of climate screens and shade nets in context. To achieve this, spectral irradiance measurements were conducted under two polytunnel and a glass greenhouse structures. We tested a selection of climate screens and shade nets readily available in North America and commonly used in horticultural applications. More specifically, we compared the effects of different patterns, shading factors, and colours of climate screen and shade net on transmitted light quality under these filters. This spectral characterization of the light environments created by the nets and screens can be used to estimate the photoreceptor-mediated responses most likely to be produced by plants affecting their growth and morphology. This in turn will allow us to consider which structures would best suit specific plant, location, and environment combinations. Manufacturers, growers, horticultural consultants and researchers alike would benefit from the availability of detailed information on the spectrally-selective attenuation of solar radiation by climate screens and shade nets, allowing them to choose the most appropriate materials for their specific purposes.

## Materials and methods

We tested 24 climate-screen samples (ten Harmony-type, two Luxous-type, seven Solaro-type and six Tempa-type) and four insect-screen samples provided by Svensson (AB Ludvig Svensson, KINNA, Sweden, http://www.ludvigsvensson.com/climatescreens/products/climate-screens). Twenty-six shade-net samples (16 Sombra-type and ten Sombra raschel-type (patterned)) and 12 thrip, aphid, and insect -net samples (two Anti-Trip-type, eight Anti-Afidos-type and two Anti-Insect types) were provided by Mallas Textiles (Mallas Textiles Fabricantes, Chimalhuacán, Mexico, https://www.mallastextilesfabricantes.com/productos). Detailed properties of the screens and nets are given in S1 Table and S2 Table.

Solar spectral photon irradiance (μmol m^-2^ s^-1^) transmitted by the materials was measured with an array spectroradiometer, which had been calibrated for measurements of UV and visible solar radiation (Maya2000 Pro Ocean Optics, Dunedin, FL, USA; D7-H-SMA cosine diffuser, Bentham Instruments Ltd, Reading, UK). A protocol of dark measurements and measurements excluding UV radiation was followed to quantify and account for the dark noise and stray light in the UV waveband. Both a correction for the shape of the slit function and for stray light were included in the post-processing of the spectra [23–25].

In order to place our measurements of climate screens and shade nets in context, spectral irradiance measurements were conducted in two polytunnels and one glass greenhouse: (1) a tunnel structure 6 x 31 m, height 2.8 m, covered with polyethylene (polytunnel) (SunMaster, Lumite Inc., Georgia, USA) installed 9 months before the first measurements; (2) a greenhouse structure 13 x 13 m, height 3.5 m, covered with two laminated layers of tempered safety glass 6-mm thick (Guardian Industries, Carleton, Michigan, USA), installed in 2002 and (3) outdoors; (4) another polyethylene tunnel 7 x 35 m, height 3.3 m, made from Klerk’s K-50 Clear polyethylene (RKW North America, Kentucky, USA) installed 7 months before the measurements. The cosine diffuser was held exactly horizontal to the ground on a wooden plate on a tripod at a height of 1.4 m above the ground. The measurements were taken along a transect of 12–15 points inside the tunnels and the greenhouse to account for any spatial variability. The measurements were done in clear sunny weather conditions in North Carolina, for (1) (2) and (3) between 10 a.m. and 2 p.m. local time on April 4^th^ (35.78°N, -78.67°W), and for (4) on March 8^th^ 2017 (35.06°N, -80.59°W).

All the measurements of irradiance under climate screens and shade and insect nets were done at NC State University campus (35.78°N, -78.67°W) between July 31 and August 10, 2017 on clear days in sunny conditions between 10 a.m. to 2 p.m. local time. The measurements were done in an open field with no surrounding structures or buildings within ~20 m. Repeated measurements of each different sample were made in a randomised order, thus ensuring comparability among measurements. The same measurement protocol as described above was followed except that the diffusor on the tripod was 0.7 m above the ground, and the sample was secured to a wooden plate just above but not touching the diffusor. A test comparing four larger (1 x 1 m) samples against those of the standard dimensions that we used, found that the area of screen/net measured did not affect the results. Thus, there was no evidence that the relatively small dimensions of the sample, allowed unfiltered diffuse or scattered radiation to be measured. Measurements under each screen/net sample (Svensson 13 x 19 cm, Mallas Textiles 8 x 10 cm) were made twice to account for any possible effect of sample placement over the cosine diffuser and change in the sun angle during a set of measurements. In our measurements and analyses we focused on the differences in spectral quality, created when employing these screens and nets, in order to address the lack of detailed studies of these light environments. Other radiometric properties of these materials, like reflectance and thermal characteristics, are already adequately described e.g. by Nijskens et al. and Cascone et al. [26,27].

Measurements of solar spectral irradiance in the wavelength range from 290 nm to 900 nm were processed in R [28], using the *photobiology* packages developed for spectral analysis [29]. We present spectral photon irradiance (μmol m^-2^ s^-1^) which is more relevant than energy irradiance (W m^-2^) when studying plants. A plant absorbs photons producing a chemical change (Grotthus Law). Nevertheless, essentially the patterns of spectral attenuation by different screens and nets will be consistent, irrespective of whether spectra are expressed as photon or energy irradiance.

Utilizing predefined functions available in the *photobiology* packages, we calculated the integrals and ratios (of these integrals) as follows:: UVB:PAR 280–315 nm/400-700 nm, UVA:PAR 315–400 nm/400-700 nm, blue:green (B:G) 420–490 nm/500-570 nm, blue:red (B:R) 420–490 nm/620-680 nm. Red and far-red for the calculation of R:FR ratio are 655–665 nm and 725–735 nm, respectively. UVB radiation and UVA radiation are defined according to ISO [30], blue, green and red according to Sellaro et al. [31], and R:FR according to Smith [32]. A common approach used in horticulture to compare light sources and experiments is to assess the relative contributions of different wavebands between 400 nm to 900 nm by dividing them into 100-nm increments [33]. We also used this approach giving, blue100 = 400–500 nm, green100 = 500–600 nm, red100 = 600–700 nm, far-red100 = 700–800 nm and near-infrared100 = 800–900 nm. The same definitions of the UV-waveband are maintained for both spectral integrals and their ratios throughout, i.e. according to ISO. This is because the UVB and UVA wavebands of solar radiation follow distinct daily patterns of variation; UVB irradiance is highest during the four hours around solar noon, whereas the UVA waveband of solar radiation remains a similar proportion of total irradiance throughout the day. These differences also imply that UVA and UVB radiation follow different diurnal and seasonal patterns of variation [34]. A nominal shading factor (SF) was used in the analysis of the results and figures to demonstrate that reduction in total irradiance due to the depth of shade does not, in itself, affect the spectral quality under the screens and nets.

The solar irradiance varies even during clear sunny days according to solar elevation. During the period when we measured between 10 a.m. and 2 p.m. the total changes in irradiance can be large, but the relative changes in spectral composition are small (i.e. negligible changes in the relative proportion of different wavebands). These patterns are confirmed by the results of radiative transfer modelling of spectral irradiance for each occasion and location where we measured (S3 Table). For example, on one of our measurement dates, August 10^th^, the UVB irradiance varied between 2.43 μmol m^-2^ s^-1^ at 10 a.m. and 5.65 μmol m^-2^ s^-1^ at solar noon, and the blue irradiance varied between 388.7 μmol m^-2^ s^-1^ and 579.2 μmol m^-2^ s^-1^, respectively. However, the percentage contributed to total photon irradiance (280–900 nm) by the UVB waveband only changed from 0.10% to 0.16% and by the blue waveband from 16.42% to 16.59% over this period. To make our results as widely useful as possible, both within this study and with other studies, we report the spectral composition (Tables 1, 2 and 3) rather than the values of irradiance (given in S3 Table) in the main body of the manuscript.

**Table 1:** Percentage of total photon irradiance (280–900 nm) in each waveband measured outdoors, in a glass greenhouse, and two types of polytunnel.

Type	Material	UVB 280–315 nm	UVA 315–400 nm	Blue 400–500 nm	Green 500–600 nm	Red 600–700 nm	Far-red 700–800 nm	NIR 800–900 nm
Glass	Glass	0.002	3.2	15.9	21.7	22.4	19.6	17.3
K-50 Clear	Polyethylene	0.048	3.0	13.5	19.6	21.8	21.4	20.7
SunMaster	Polyethylene	0.002	1.3	13.8	20.2	22.5	21.6	20.6
Ambient sunlight/outdoors		0.082	4.3	15.5	20.0	21.0	20.0	19.1

**Table 2:** Different Svensson climate screen samples and their percentage transmittance of integrated total spectral photon irradiance 280–900 nm. More details about the properties of the screens are given in S1 Table.

Type	Type number	Material	Nominal shading factor*	UVB 280–315 nm	UVA 315–400 nm	Blue 400–500 nm	Green 500–600 nm	Red 600–700 nm	Far-red 700–800 nm	NIR 800–900 nm	Recommended use**
Harmony	3015	polyolefine	30	0.062	3.5	14.7	19.2	20.4	20.9	21.3	High light diffusion, more even light distribution, increased crop quality due to lower temperature of the fruit or flower.
	3315	polyolefine-polyester	33	0.029	2.0	14.4	19.9	21.5	21.2	20.9	
	3647	polyester	36	0.005	0.8	13.6	19.4	21.4	22.2	22.6	
	3915	polyolefine	39	0.055	2.8	13.9	19.0	20.6	21.4	22.3	
	4215	polyester	42	0.042	2.1	14.1	19.5	21.0	21.4	21.8	
	4647	polyester	46	0.003	0.7	13.2	19.0	21.1	22.5	23.6	
	5120	polyolefine	51	0.051	2.6	13.6	19.2	21.2	21.5	21.8	
	5220	polyester	52	0.023	1.6	12.7	18.7	21.0	22.4	23.5	
	5747	polyester	57	0.003	0.5	11.9	17.9	20.5	23.5	25.8	
	6420	polyolefine	64	0.035	2.3	13.3	19.0	20.9	21.9	22.6	
Luxous	1347	polyester	13	0.001	1.0	15.5	20.5	21.5	21.0	20.4	Energy savings with high light transmission. Condensation forming on the screen is better absorbed.
	1547	polyester	15	0.000	1.3	15.2	20.3	21.4	21.2	20.7	
Solaro	3815	polyolefine	38	0.113	5.1	16.0	19.2	19.5	19.9	20.2	The ultimate shading due to its open structure for maximum ventilation. Can be used at night to reduce radiation losses that otherwise cause condensation on plants.
	5115	polyolefine	51	0.076	4.0	15.1	19.9	21.0	20.3	19.6	
	5120	polyolefine-aluminium	51	0.080	3.7	14.7	19.0	20.1	20.9	21.6	
	5220	polyester-aluminium	52	0.023	1.6	14.6	19.9	21.2	21.4	21.3	
	6125	polyester-aluminium	61	0.075	3.9	14.8	18.1	18.5	21.3	23.3	
	6720	polyolefin	67	0.097	4.5	15.7	19.7	20.3	20.1	19.6	
Tempa	5155	polyolefine-aluminium	51	0.071	3.7	15.1	19.7	20.6	20.6	20.2	A single-screen solution for shading, cooling and maximum energy saving. Doubles up as an effective shading screen by day. A more even temperature throughout the greenhouse when used with a pad and fan cooling system. Maximum cooling when installed above plants in a greenhouse with side ventilation.
	5557	polyester-aluminium	55	0.007	1.2	14.7	20.0	21.1	21.5	21.5	
	6360	polyo.-alumin.-polye.-modacryl	63	0.064	3.3	14.8	19.2	20.2	20.9	21.5	
	6562	polyester-aluminium	65	0.007	1.3	14.8	19.7	20.7	21.6	21.9	
	6960	polyolefine-aluminium	69	0.061	3.3	14.9	19.4	20.2	20.7	21.4	
	7567	polyester-aluminium	75	0.001	1.0	15.0	17.9	17.6	22.7	25.9	
Insect	1515	polyolefine	15	0.080	4.1	15.2	19.8	20.8	20.3	19.8	Keeping harmful insects outside the greenhouse and useful insects inside.
	1535	polyolefine-acrylic	15	0.070	3.7	15.3	19.9	20.9	20.4	19.8	
	2777	polyolefine	27	0.062	3.7	15.3	19.9	21.0	20.3	19.7	
	4045	polyolefine	40	0.058	2.7	15.1	20.1	21.2	20.7	20.2	
Ambient sunlight/outdoors				0.082	4.3	15.5	20.0	21.0	20.0	19.1	

* Svensson climate screens and Mallas Textiles shade nets are classified according to the shading factor. Direct and diffuse radiation factors are typically reported separately for Svensson climate screens. The shading factors for direct and diffuse radiation follow very similar patterns, and for clarity we present only the SF for direct radiation.

** Information taken from Svensson product brochures

**Table 3:** Mallas Textiles shade net and insect net samples and their percentage transmittance of integrated total spectral photon irradiance 280–900 nm. More details about the properties of the nets are given in S2 Table.

Type colour	Colour pattern	Nominal shading factor	UVB 280–315 nm	UVA 315–400 nm	Blue 400–500 nm	Green 500–600 nm	Red 600–700 nm	Far-red 700–800 nm	NIR 800–900 nm
**Shade net (Sombra)**									
negro	black	95	0.072	3.7	12.7	17.0	18.2	23.2	25.2
cafe	dark brown	95	0.060	3.3	12.5	17.5	20.8	22.4	23.5
cafebeige	dark brown/light brown	95	0.040	2.0	9.0	16.6	22.7	24.4	25.3
azulblanco	blue/white	95	0.019	1.1	15.1	17.7	17.0	21.4	27.8
negro	black	90	0.080	3.9	14.8	19.7	20.9	20.6	20.0
verde	green	90	0.069	3.6	14.9	20.6	19.3	20.1	21.4
ambar	amber	90	0.032	1.7	9.0	17.7	22.8	24.0	24.6
azul	blue	90	0.026	1.5	21.7	14.6	7.0	17.0	38.2
negro	black	80	0.075	4.0	15.2	20.1	21.3	20.1	19.1
verde	green	80	0.070	3.7	15.0	20.6	19.6	20.1	20.9
negro	black	70	0.081	4.1	15.4	20.2	21.4	20.0	18.9
verde	green	70	0.067	3.6	15.8	21.1	18.8	19.7	21.0
negro	black	50	0.080	4.1	15.5	20.2	21.4	19.9	18.8
bicolor	grey	50	0.064	3.5	14.8	20.1	21.5	20.4	19.6
negro	black	35	0.079	4.1	15.5	20.3	21.4	19.9	18.8
blanco	white	35	0.065	3.6	15.2	20.0	21.3	20.3	19.5
**Patterned shade net (Sombra raschel)**									
negro	black	90	0.055	3.0	12.3	17.4	19.6	22.9	24.7
verde	green	90	0.034	1.8	16.3	22.7	10.6	18.0	30.7
ambar	amber	90	0.032	1.8	9.6	17.7	22.4	23.8	24.6
azul	blue	90	0.027	1.9	20.5	16.6	9.5	18.1	33.4
negro	black	80	0.069	3.7	14.2	19.2	20.9	21.0	21.0
verde	green	80	0.023	1.7	16.4	22.8	9.9	18.0	31.2
negro	black	70	0.072	3.9	14.8	19.7	21.1	20.4	20.0
verde	green	70	0.061	3.1	16.0	21.3	16.1	19.2	24.2
negro	black	50	0.078	4.1	15.2	20.0	21.2	20.1	19.4
negro	black	35	0.083	4.2	15.3	19.9	21.1	20.0	19.3
**Anti-Thrip**		**Mesh size**							
cristal	lightgrey	24x12	0.080	4.0	15.2	19.9	20.9	20.3	19.6
cristal	lightgrey	22x12	0.083	4.1	15.3	20.0	20.9	20.2	19.4
**Anti-Aphid (Anti-Afidos)**									
cristal	lightgrey	20x10	0.083	4.1	15.3	20.0	21.1	20.1	19.3
bicolor	darkgrey	20x10	0.085	4.1	15.3	20.1	21.2	20.1	19.1
negro	black	16x16	0.085	4.0	15.1	20.0	21.3	20.2	19.3
ambar	amber	16x16	0.062	3.1	13.0	19.5	22.0	21.4	20.9
negro	black	16x10	0.087	4.1	15.4	20.2	21.4	19.9	18.8
ambar	amber	16x10	0.062	3.0	12.5	19.5	22.2	21.7	21.2
cristal	lightgrey	16x10	0.083	4.1	15.4	20.1	21.2	20.1	19.2
bicolor	darkgrey	16x10	0.082	4.1	15.3	20.1	21.2	20.1	19.2
**Anti-Insect (Anti-Insectos)**									
cristal	lightgrey	10x10	0.087	4.2	15.5	20.2	21.2	20.0	19.0
bicolor	darkgrey	10x10	0.089	4.2	15.5	20.2	21.3	19.9	18.9
**Ambient sunlight/outdoors**			0.082	4.3	15.5	20.0	21.0	20.0	19.1

Modelled solar radiation spectra (S3 Table) at each measurement location were calculated with library of radiative transfer programs, *libRadtran*, version 2.0.1. [35] using the radiative transfer equation solver DISORT. Simulations for 15-min intervals are presented following Lindfors et al. [36], slightly modified to produce spectra of 280–900 nm. Required inputs were pyranometer measurements of global radiation, total ozone column data, column integrated water vapour data and surface types defined by the International Geosphere Biosphere Programme (IGBP).

## Results

**Fig 2:**
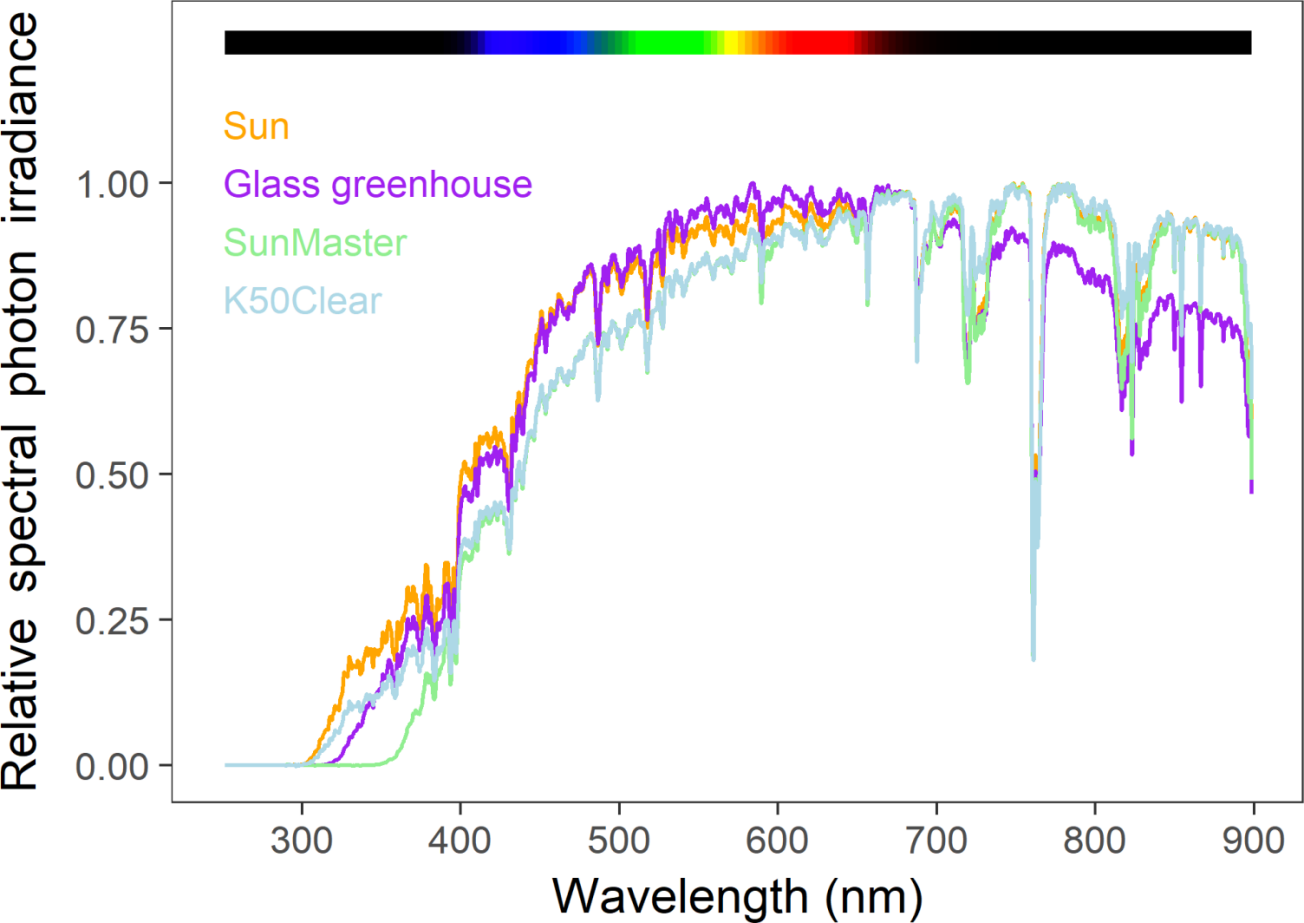
Spectral photon irradiance outdoors, in a glass greenhouse, and two types of polytunnel. To assist in their comparison, each spectrum is normalized to 1 at the wavelength of its maximum spectral irradiance.

**Fig 3:**
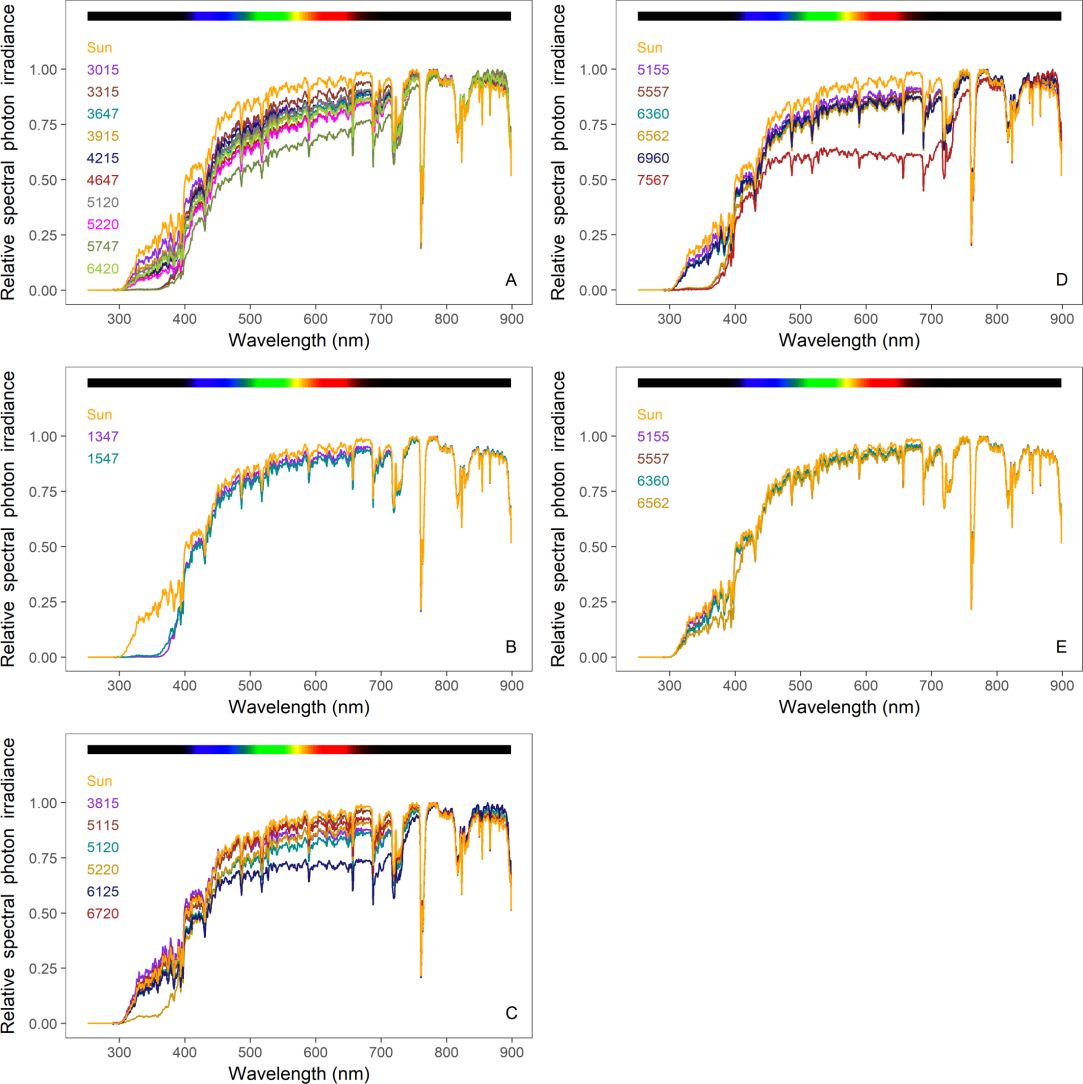
Spectral photon irradiance under Svensson climate screen samples in ambient sunlight. (A) Harmony; (B) Luxous; (C) Solaro; (D) Tempa; (E) Insect screens. The orange line in each panel represents the spectrum of full sunlight at the time and location of measurement. To assist in their comparison, each spectrum is normalized to 1 at the wavelength of its maximum spectral irradiance.

**Fig 4:**
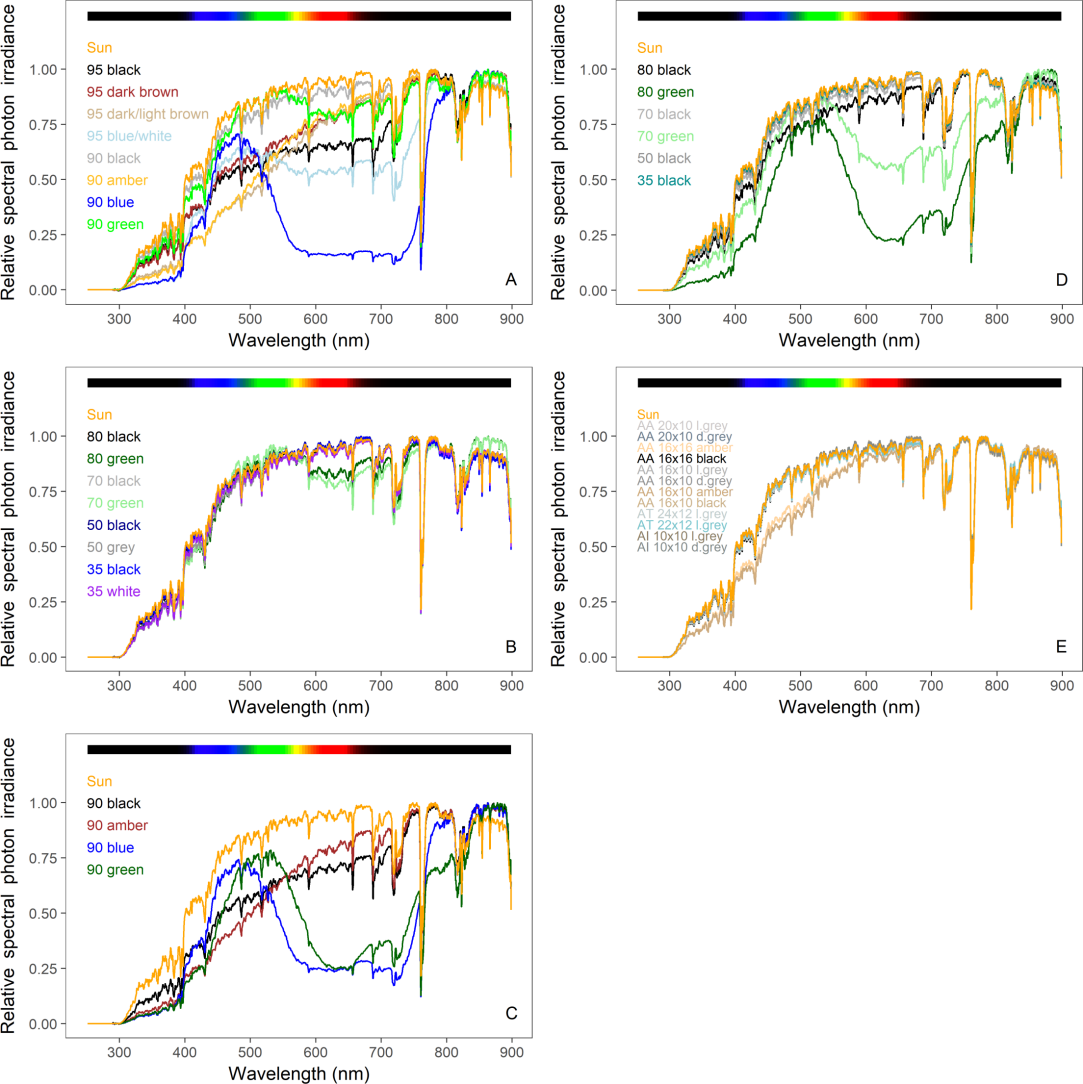
Spectral irradiances under Mallas Textiles shade and insect net samples in ambient sunlight. (A) Shade net nominal shading factor 90–95; (B) Shade net nominal shading factor 35–80; (C) Patterned shade net nominal shading factor 90; (D) Patterned shade net nominal shading factor 35–80; (E) Insect nets with varying mesh sizes and colours. For the insect nets, AA refers to type Anti-Afidos, AT to Anti-Trip and AI to Anti-Insectos. The orange line in each panel represents the spectrum of full sunlight at the time and location of measurement. To assist in their comparison, each spectrum is normalized to 1 at the wavelength of its maximum spectral irradiance.

**Fig 5:**
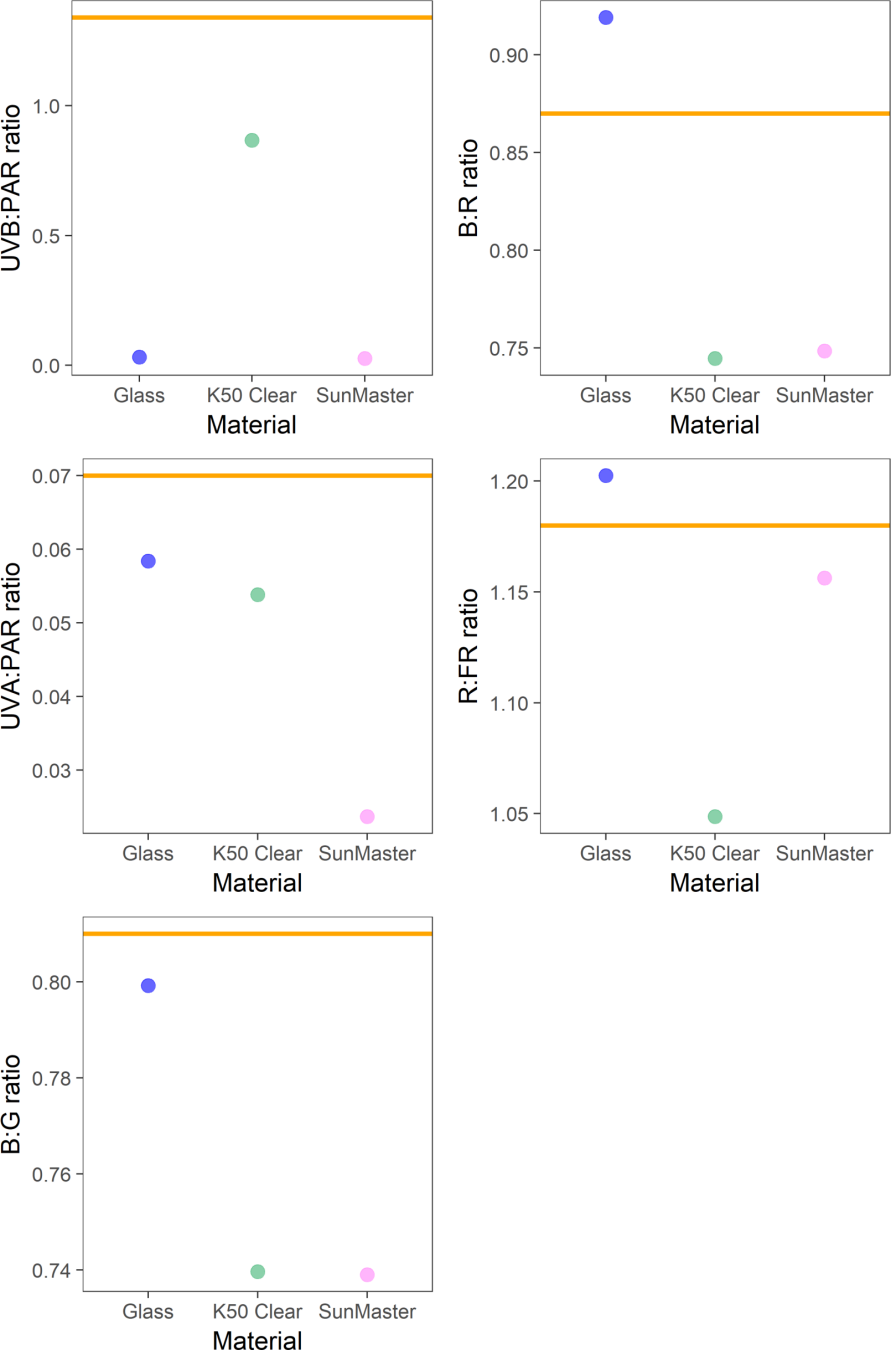
Spectral photon ratios measured in a glass greenhouse and two types of polytunnel. UVB:PAR is x1000 for ease of comparison. Points are the mean of two measurements. The orange horizontal line in each panel represents the spectral photon ratio of full sunlight at the time and location of measurement.

**Fig 6:**
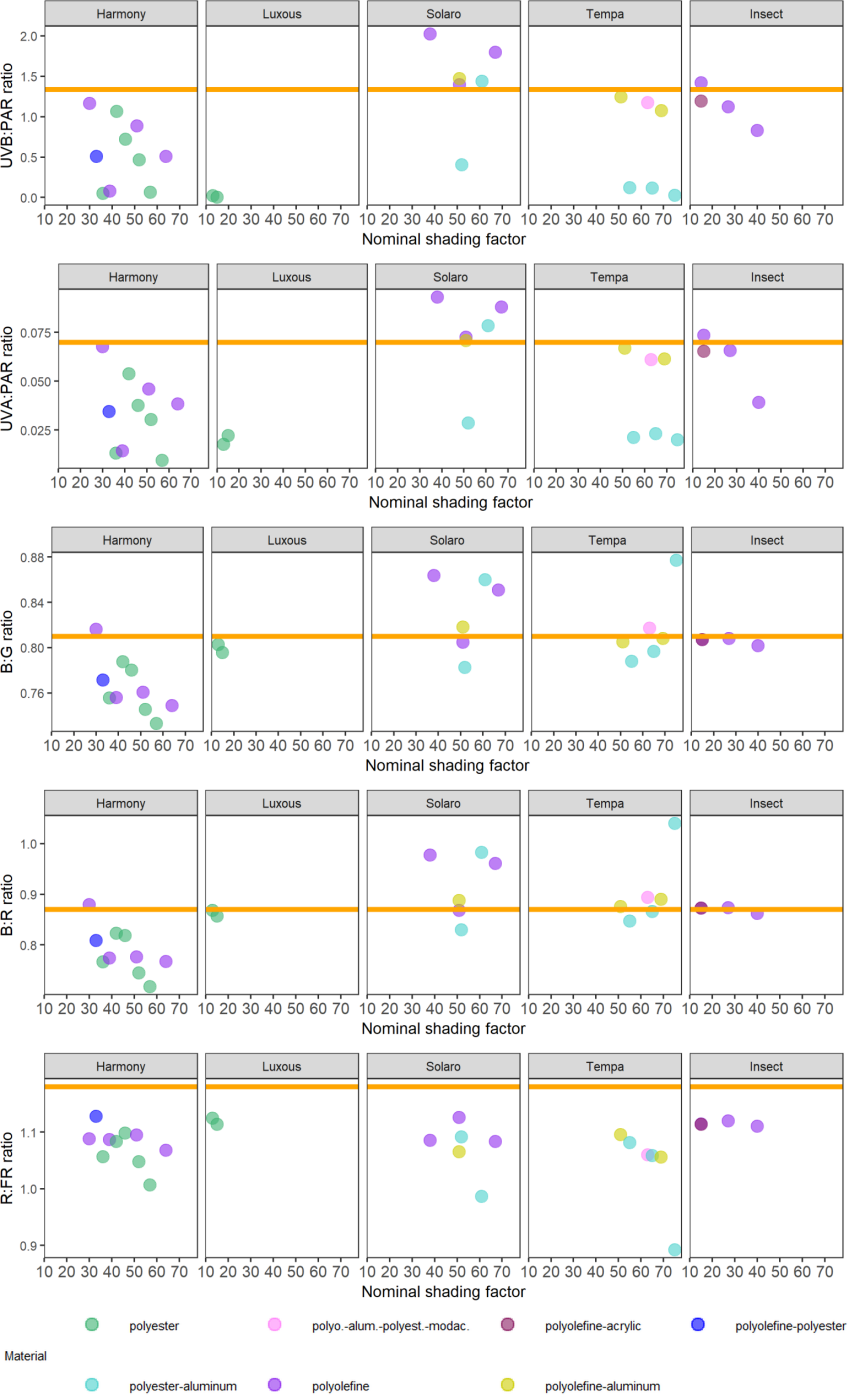
Spectral photon ratios measured under Svensson climate and insect screens in ambient sunlight. UVB:PAR is x1000 for ease of comparison. Points are the mean of two measurements. Nominal shading factors and material type descriptions are taken from manufacturer’s product brochure. The orange horizontal line in each graph represents the spectral photon ratio of full sunlight at the time and location of measurement.

**Fig 7:**
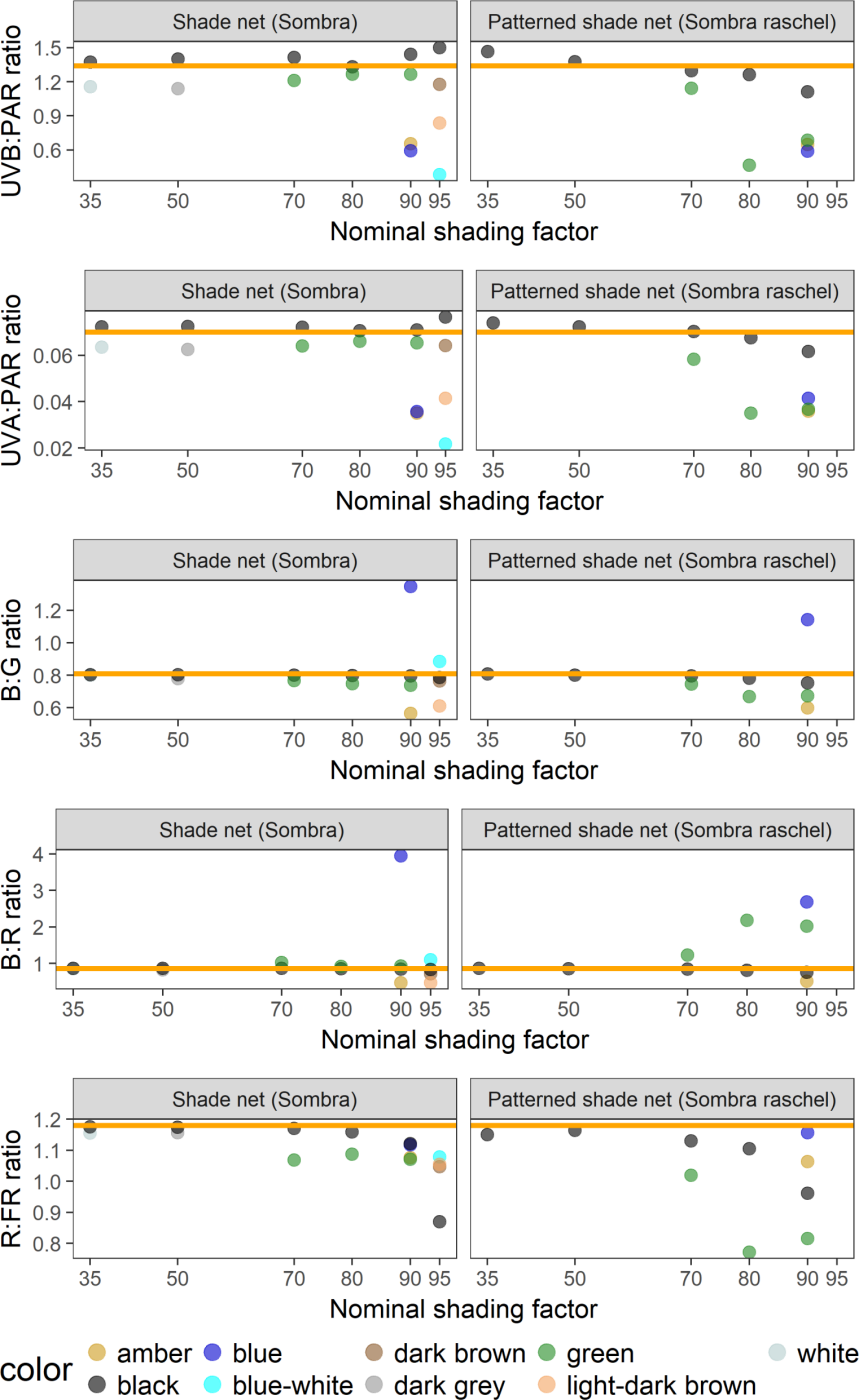
Spectral photon ratios measured under Mallas Textiles shade nets in ambient sunlight. UVB:PAR is x1000 for ease of comparison. Points are the mean of two measurements. Nominal shading factors are from the manufacturer’s product brochure. The orange horizontal line in each graph represents the spectral photon ratio of full sunlight at the time and location of measurement.

**Fig 8:**
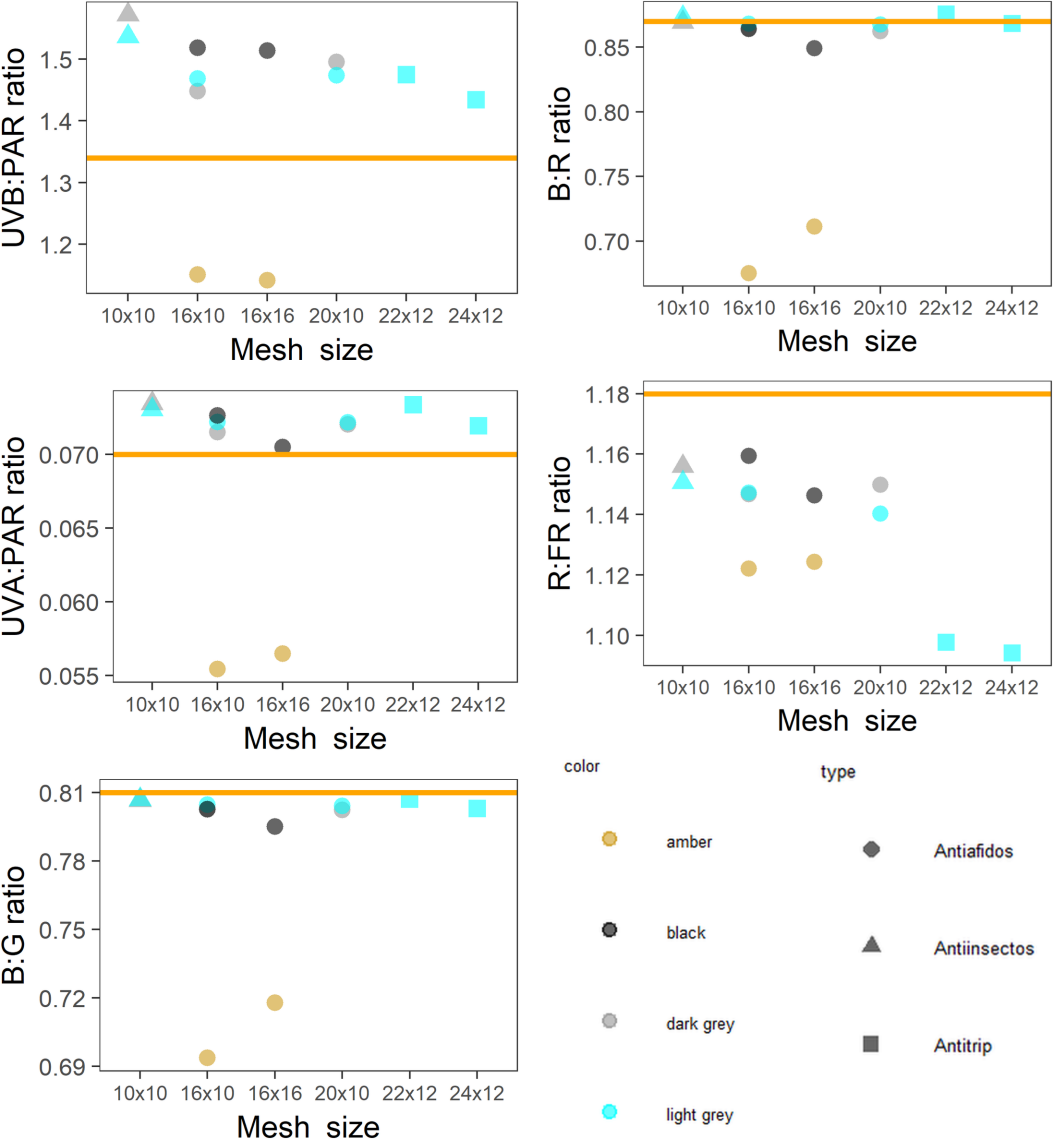
Spectral photon ratios measured under Mallas Textiles insect nets in ambient sunlight. UVB:PAR is x1000 for ease of comparison. Each point is the mean of two measurements. Mesh sizes are taken from manufacturer’s product brochure. The orange horizontal line in each graph represents the spectral photon ratio of full sunlight at the time and location of measurement.

We present our results in three different ways. The composition of spectral irradiance (280–900 nm) is presented as the relative % contribution of each waveband to the total irradiance (Tables 1, 2 and 3). To visualise these comparisons of the screens and nets, their transmittance normalized to 1 are shown in Figs 2–4, and spectral photon ratios Figs 5–8.

Spectral irradiances measured outdoors, in a glass greenhouse, and two types of polytunnel (SunMaster and K50 Clear), are presented in Fig 2 and these data are given as percentages of the full-sun spectral photon irradiance in Table 1. These comparisons reveal that the glass greenhouse effectively blocked all UVB radiation, but enriched the relative proportion of green and red wavebands by greater attenuation of other wavebands, compared to ambient sunlight. The radiation environment in the polytunnel showed that plastic K50 Clear transmits UVA radiation at a similar extent to glass, but it also allowed some UVB radiation to be transmitted. K50 Clear plastic also reduced the proportion of blue100 relative to the proportion of far-red100 and near-infrared100, compared to the composition in ambient sunlight. The other polytunnel plastic that we tested, SunMaster, fully attenuated all UVB radiation and the majority of UVA radiation as well as reducing the proportion of blue100 and enriching the proportion of red100, far-red100 and near-infrared100, compared to the composition of these wavebands in ambient sunlight.

Svensson’s climate and insect screens are pictured in the S1 Fig, and the spectral photon irradiance that they transmit are given in Fig 3A–3E with the percentages of the integrated photon irradiance (280–900 nm) in each specific waveband illustrating how the spectral composition is affected by the screens in Table 2. The Harmony-type climate screens produced the greatest variation in transmittance across different wavebands (Fig 3A). The contribution of the UVB waveband to the integrated photon irradiance varied from 0.003 to 0.062%, and that of the UVA waveband varied from 0.5 to 3.5% across all Harmony-type climate screens. Harmony-type screens 3647, 4647 and 5747, with the lowest transmittance values in the UV wavebands, created conditions that were severely depleted in UV radiation compared with ambient solar radiation (where UVB radiation comprises 0.082% of the integrated photon irradiance i and UVA radiation 4.3%) (Table 2). Further, all the Harmony-type screens changed the spectral quality by reducing blue (blue100) (11.9 to 14.7%) compared with its contribution to ambient sunlight (15.5%). The spectral composition produced by Harmony screens in wavebands other than blue was similar to that of ambient sunlight with the exceptionof Harmony 5747 (Table 2). Luxous screens entirely attenuated UVB radiation and UVA radiation below 350 nm and most of UVA radiation from 350 to 400 nm (Fig 3B), but produced a spectral composition of other wavebands that was similar to ambient sunlight (Table 2). Solaro 5220 attenuated most UVB radiation and UVA radiation, but otherwise under the Solaro screens the spectral conditions were not much different compared to the ambient sunlight (Fig 3C, Table 2). Tempa screens 6562 and 7567 entirely attenuated the UV radiation below 350 nm and most UVA radiation from 350 to 400 nm, Tempa 7567 also partially attenuated green100 and red100 (17.9 and 17.6%, compared with ambient at 20.0 and 21.0%, respectively (Fig 3D, Table 2). Other Tempa screens did not alter spectral transmittance drastically (Fig 3D, Table 2). Svensson insect screens did not change spectral quality, except 4045 which attenuated more of the UV radiation than the other Svensson insect screens, 0.058% of the integrated photon irradiance was contributed by the UVB waveband and 2.7% by the UVA waveband, compared to 0.082% and 4.3%, respectively in ambient sunlight (Fig 3E, Table 2).

The properties of the Mallas Textiles shade and insect nets that we measured are pictured in the S1 Fig and the spectral photon irradiance that they transmit are given in Table 3, with the percentages of the integrated photon irradiance in each specific waveband illustrating how the spectral composition is affected by the nets in Fig 4A–4E and Table 3. Of the shade-type (Sombra) nets with nominal shading factors (SF) of 90 and 95, nets with brown hues (dark brown, dark/light brown and amber), caused the largest decrease in the contribution of wavelengths below 500 nm to integrated photon irradiance compared with ambient sunlight (Fig 4A, Table 3). More precisely, contribution to photon irradiance from the UVB waveband was reduced to 0.032% from 0.082% in ambient sunlight; and the contribution of UVA radiation was reduced to 1.7% from 4.3% in ambient sunlight. Black shade nets (Sombra) with SF of 35, 50, 70 and 80 did not change the light quality compared to ambient sunlight, but those with the highest SF of 95 produced the greatest relative contribution of longer wavelengths beyond 700 nm to photon irradiance (far-red100 23.2% compared with ambient 20.0% and NIR100 25.2% compared with the ambient 19.1%) (Fig 4A and 4B, Table 3). The two blue or blue-white nets reduced the relative contribution of the UV-wavebands and green100 and red100 the most, and enriched the relative contribution from the NIR waveband; a high NIR transmittance relative to short wavebands is characteristic of all those nets with high shading factors, SF 90 or SF 95 (Table 3). The spectral quality was similar under each of the three green shade nets with different SF (70, 80 and 90), but transmittance of the UV and red100 wavebands was low compared with ambient sunlight (Fig 4B, Table 3). White and grey shade nets did not change the composition of spectral irradiance greatly compared with ambient sunlight (Fig 4B, Table 3).

Similarly to black-coloured shade nets (Sombra), patterned nets (Sombra raschel) with SFs of 35, 50, 70 and 80 did not change the light quality compared to ambient sunlight (Fig 4C and 4D, Table 3). In contrast to green shade nets, green patterned nets increased the relative contribution of wavelengths beyond 800 nm to the integrated photon irradiance, irrespective of the SF, but both types of green net similarly reduced irradiance in the red waveband (Fig 4C and 4D, Table 3). Blue and amber coloured patterned nets (SF 90) produced similar changes to spectral quality to the respectively coloured shade nets described above (Fig 4C, Table 3).

Similarly to Svensson insect screens and white and grey coloured Mallas Textiles shade nets, Mallas Textiles insect nets had only small effects on the spectral quality of irradiance that they transmitted, with the exception of amber nets, that performed similarly to shade and patterned nets with brown hues in decreasing the relative contribution of wavelengths below 500 nm to the integrated photon irradiance compared with that of ambient sunlight (Fig 4E, Table 3).

Spectral photon ratios calculated from spectral irradiance measured in a glass greenhouse and two polytunnels are presented in Fig 5. The attenuation of UVB radiation by glass led to a UVB:PAR ratio in the greenhouse that differed most from ambient sunlight, whereas the UVB:PAR and UVA:PAR ratios under K50 Clear plastic were relatively similar to those measured in ambient sunlight. However the B:G, B:R and R:FR ratios in the K50 Clear polytunnel differed more from ambient sunlight than did those produced by the glass greenhouse (Fig 5). Comparing the three materials, the light environment in the SunMaster polytunnel differed most from ambient sunlight, as reflected in the spectral photon ratios of wavebands (Fig 5).

Spectral photon ratios calculated from spectral irradiance measured under Svensson climate and insect screens were plotted against nominal shading factors (Fig 6). Insect and Luxous screens produced the most similar values to ambient sunlight for these ratios of all the nets and screens tested, with the exception of the UVB:PAR and UVA:PAR ratios measured under the Luxous screens which were much lower than ambient sunlight (Fig 6). Harmony-type screens produced a light environment with consistently lower spectral photon ratios, i.e. less irradiance in the shorter wavebands, than ambient sunlight, whereas the Solaro-type screens tended to produce higher spectral photon ratios than ambient sunlight and Tempa-type screens varied according to their composition.

Spectral photon ratios calculated from spectral irradiance measured under Mallas Textiles shade nets and patterned shade nets show that net colour had a strong effect on the spectral quality (Fig 7). In particular, blue, amber and green nets created light conditions that differed most from ambient sunlight. Black and brown coloured nets created more neutral shade, i.e. of similar spectral quality to sunlight, but amber-coloured nets produced consistently lower spectral photon ratios than ambient sunlight. When the spectral photon rations under Mallas Textiles insect nets were plotted against the mesh size, the amber-coloured nets differed markedly from the other nets irrespective of mesh size (Fig 8). The relationship between mesh size and spectral photon ratio also illustrates the large difference in R:FR ratio of Anti-Trip nets of the largest mesh sizes (Fig 8).

## Discussion

The irradiance and spectral quality in the greenhouse and the two polytunnels differed in the shortest wavebands, i.e. UVB, UVA and blue wavebands compared with ambient sunlight. For wavelengths above 700 nm, the normalised spectrum of the two plastics gave a relatively high transmittance, whereas the glass gave a relatively low transmittance compared with ambient sunlight. Concerning the climate and insect screens and shade and insect nets, variation in the spectral quality was high under the shade nets compared with the other materials. The following discussion examines the changes in spectral composition in specific wavebands and spectral photon ratios produced by these screens and nets, in the context of their possible consequences for plant growth and morphology.

### Attenuation of UVB and UVA radiation by screens and nets and its implications

Of the four different types of Svensson climate screens, the Harmony-type screens were found to decrease the percent of UVB radiation, UVA radiation, and blue light compared with longer wavelengths. Luxous- and Tempa-type screens, and one Solaro screen, are all made from polyester and aluminium. Since polyester attenuates UVB radiation, it is unsurprising that these screens produced much lower UVB:PAR and UVA:PAR ratios than are found in ambient full solar radiation. Concerning the Mallas Textiles shade nets, all but the black nets decreased the proportion of UVB and UVA radiation in the transmitted spectrum, and majority of nets but in particular those with a brown hue (dark/light brown and amber) also decreased the proportion of blue light. Notably, the net pattern itself can affect the transmitted light quality even when nets are of similar colour: for instance the two shade nets and patterned-shade nets in green (which had same nominal shading factor of 90) produced different UVB:PAR and UVA:PAR ratios; the shade net produced a UVB:PAR ratio of 1.3 and a UVA:PAR ratio of 0.065 and for the patterned-shade net these values were 0.70 and 0.037, respectively. The mesh in shade nets is more tightly and regularly woven than that of the patterned-shade nets (S1 File), and this could change the proportion of diffuse to direct radiation beneath which might partly explain the difference in spectral irradiance beneath them. Taken together, five Solaro-type climate screens, five black shade net and two black patterned shade nets produced high UVB:PAR and UVA:PAR ratios, possibly creating light conditions whereby coordinated photoreceptor-mediated responses would produce a different effect on plant growth than under ambient sunlight or a net with a lower UV:PAR ratio.

To the best of our knowledge, there are no studies of the UVB:PAR or UVA:PAR ratios nor plant responses to these ratios in horticultural environments. Even studies merely concerned with the effect of UV radiation on the growth of horticultural crops are scarce. However, Wargent et al. [37] found differences in lettuce (*Lactuca sativa*) plants grown in a polytunnel transmitting UVB-radiation and one equipped with UVB-attenuating plastic. Higher photosynthetic rates were measured in plants grown in the presence of UV radiation and the relative growth rate of plants pre-acclimated to UV radiation also increased. When transplanted to a uniform field environment, those plants initially propagated in the light environment which included UVB radiation had a higher harvestable yield than those originating from the UVB-depleted environment. This suggests that the presence of UVB radiation in the controlled environment conferred seedlings with an improved capacity to withstand transplant shock. In all, the role of UVB signalling in various crop production scenarios should be further studied, given its potential to be utilized in the hardening of seedlings to outdoor conditions. Additionally, UV radiation is implicated in the accumulation of plant secondary metabolites (phenolic compounds, including flavonoids etc.), that are associated with plant colour, taste and perceived health-promoting attributes [38]. In a relevant horticultural context, pea plants can regulate the accumulation of specific flavonoid compounds contingent on whether they are grown under UV-radiation or blue light [39].

### Attenuation of blue, green and red light by screens and nets and its implications

Sellaro et al. [31] measured midday solar irradiance in the field beneath different plant canopies, litter or soil layers, or exposed to unfiltered sunlight. They found changes in the B:G ratio to be decoupled from those of blue light, signifying that under-canopy green light was driving the variation in spectral quality across these wavebands. With this as a starting point, the authors examined hypocotyl length of seedlings of model plant *Arabidopsis thaliana* grown under controlled conditions in B:G ratios and irradiances within the range of ratios they measured in natural environments. They measured shorter hypocotyl length under the B:G ratio of 1.1 compared with the other 0.5. The authors concluded that the B:G ratio may provide information to plants and about the degree of shading by neighbours independently from the R:FR ratio [31]. A similar conclusion was drawn from a fully-factorial experiment with *A*. *thaliana* exposed to a high and low R:FR ratio with or without supplemental green light, where the addition of green light enhanced the low R:FR response [40]. With respect to the climate screens that we tested, the Svensson screens had B:G ratios relatively close to that of ambient sunlight, while one Tempa screen made from polyester-aluminium produced a higher B:G ratio than ambient sunlight. For Mallas Textiles shade nets, B:G was lower for amber-coloured ones, and higher for blue. In addition, B:G ratio was lower than ambient under the amber coloured insect nets. Given these differences, there is a clear need for studies of the B:G ratio and plant responses to these ratios in horticultural environments.

Hernandez and Kubota [41] reported that varying the proportion of blue and red light, determined how high and low solar daily light integral (DLI) treatments affected cucumber seedling growth in a greenhouse experiment. In each solar DLI treatment, the proportion of blue100 and red100 wavebands was manipulated. The percentage blue100 was either 0%, 4% or 16%. Under the higher DLI (16.2 mol m^-2^ d^-1^) there were no significant differences in cucumber growth under the contrasting blue-red light combinations. Whereas, under the lower DLI (5.2 mol m^-2^ d^-1^), leaf number, leaf area and seedling dry mass all decreased with proportionally more blue light in the treatment. This indicates that shade nets producing a higher nominal shading factor and low B:R spectral photon ratio, with low % blue light, may produce greater differences in plant morphology and growth, typical of the shade avoidance syndrome (SAS), than shade nets with a lower nominal shading factor but higher B:R spectral photon ratio.

### Modification of the R:FR ratio by screens and nets and its implications

Demotes-Mainard et al. [42] comprehensively review R:FR-related responses of plants in a horticultural context. They compile the effects of low R:FR and reduced expression of PHYB on plant vegetative development, flowering, photosynthesis and pathogen–pest–drought tolerance. Arthurs et al. [6] measured light quality for the duration of a whole year under structures with red, blue, pearl and black nets (all ChromatiNet®) using an optical UV/VIS spectrometer. They found that R:FR ratio (defined in their study as 600–700 nm/700-800 nm) was similar to that in ambient sunlight under their black net, and slightly reduced under their blue net. In the present study, Harmony-type screens kept the spectral photon ratios of R:FR below that of full sunlight, whereas Solaro and Tempa produced higher R:FR ratios than ambient sunlight. Insect and Luxous screens gave spectral quality similar to ambient sunlight, except in the UV-waveband. All screens had a R:FR ratio only slightly below that measured in sunlight, but not to the extent that they would create conditions that could be regarded as equivalent to plant canopy shade. Concerning the shade nets, the lowest R:FR ratios of around 0.8 were produced under the green patterned net, a value that is equivalent to light canopy shade and could potentially enhance stem and internode elongation in certain species [31].

### Use of spectral characterization under screens and nets to interpret differences in plant performance

Ilíc and Fallik recently reviewed studies of plant physiological responses to light condition under different coloured shade nets [43], reporting effects on yield and quality parameters and phytochemical contents of vegetables such as tomatoes and lettuce and herbs, both at harvest and after storage. Our study provides detailed information about solar irradiance and its spectral transmittance through a broad selection of climate screens and shade and insect nets. We propose that such spectral characterization should be applied to studies measuring plant growth and morphological responses under different types of screens and nets. This type of comparison should help to reconcile plant responses that might otherwise appear contradictory, such as those presented by Castronuovo et al. [44]. There is a need for standardized methods of reporting on and comparing climate screens and shade and insect nets used in horticultural applications and the spectral irradiance in the light environments that they create. This would facilitate direct comparisons between experiments and plant species/varieties, whereas merely reporting shading factors and using inconsistent definitions for wavebands does not allow generalisations to be made about plant performance. Manufacturers, growers, horticultural consultants and researchers alike would benefit from the availability of detailed information when selecting the most appropriate materials for climate screens and shade nets.

## Conclusions

The effect on solar radiation of climate screens and shade nets used in horticultural applications is traditionally described using attributes related to the transmittance of PAR and for insect nets to its mesh size. Our study shows that the spectral composition (light quality) transmitted by these screens and nets is affected by their material properties. Selection of appropriate materials for the desired use, whether in a commercial or research greenhouse or polytunnel, requires consideration of the required spectral composition as well as total reduction in irradiance they cause. Comparability among trials and experiments in these controlled environments calls for standardized methods of comparing and reporting on the screens and nets used to account for differences in the light environment that they create.

## Supporting information

S1 Table Svensson climate screen and insect screen properties. (DOCX)

S2 Table Mallas Textiles shade net and insect net properties. (DOCX)

S3 Table Spectral photon irradiance and percentage of total photon irradiance in each waveband calculated from simulated irradiances. (DOCX)

S1 Fig Pictures of Svensson climate and insect screens and Mallas Textiles shade nets and insect nets. (PDF)
