# Supplementary material for: Light quality characterization under climate screens and shade nets for controlled-environment agriculture
Source: PLoS One. 2018 Jun 25;13(6):e0199628. doi: 10.1371/journal.pone.0199628 (PMC6016941; doi:10.1371/journal.pone.0199628)
Supplement: S1 Table — (DOCX) [file pone.0199628.s001.docx]

**S1 Table A. Svensson climate screen properties, all available information taken from the product sheets provided by the manufacturer. * The first value for the shading factor (PAR) is measured with an integrating sphere, the second one, where available, according to the standard protocol GTB 1252 2012 of WUR. Energy saving percentage defined by Svensson method, UV transmission measurement protocol not defined by the manufacturer. Main function of each type: Harmony = high grade light diffusion, Luxous = energy saving with maximum light transmission, Solaro = ultimate solar protection, Tempa = dual thermal control.**

| **Type** | **Type no.** | **Material** | **Pattern** | **Weight  gm^-2^** | **Width of strips mm** | **Nominal shading factor % direct*** | **Nominal shading factor % diffuse*** | **Energy saving %** |
| --- | --- | --- | --- | --- | --- | --- | --- | --- |
| Harmony | 3015 | polyolefin | open2-whitediffuse1-open2-whitediffuse1-open1-whitediffuse1 | 115 | 4 | 30/38 | 37/43 | 15 |
|  | 3315 | polyolefin-polyester | whitediffuse1-transparent1-open1-whitediffuse1-open1-transparent1-whitediffuse1-open1 | 51 | 4 | 33/40 | 39/46 | 15 |
|  | 3647 | polyester | transparentdiffuse3-whitediffuse1-transparentdiffuse2-whitediffuse1 | 57 | 4 | 36/43 | 44/50 | 47 |
|  | 3915 | polyolefin | whitediffuse1-open1 | 120 | 4 | 39/48 | 45/52 | 15 |
|  | 4215 | polyester | whitediffuse2-open1-whitediffuse1-transparentdiffuse1-open1 | 54 | 4 | 42/48 | 46/53 | 15 |
|  | 4647 | polyester | whitediffuse1-transparentdiffuse1 | 61 | 4 | 46/54 | 53/59 | 47 |
|  | 5120 | polyolefin | whitediffuse2-open1 | 123 | 4 | 51/57 | 54/61 | 20 |
|  | 5220 | polyester | whitediffuse3-open1-whitediffuse2-open1 | 60 | 4 | 52/59 | 56/63 | 20 |
|  | 5747 | polyester | whitediffuse3-transparentdiffuse1-whitediffuse2-transparentdiffuse1 | 65 | 4 | 57/63 | 62/67 | 47 |
|  | 6420 | polyolefin | whitediffuse1-whiteblack1-whitediffuse1-open1 | 134 | 4 | 64/71 | 66/69 | 20 |
| Luxous | 1347 | polyester | transparent full | 58 | 4 | 13/- | 20/- | 47 |
|  | 1547 | polyester | transparent  diffuse full | 51 | 4 | 15/- | 24/- | 47 |
| Solaro | 3815 | polyolefin | whiteblack1-open2 | 100 | 4 | 38/39 | 41/46 | 15 |
|  | 5115 | polyolefin | whiteblack1-open1 | 110 | 4 | 51/55 | 55/59 | 15 |
|  | 5120 | polyolefin-aluminium | aluminum1-open1 | 182 | 4.6 | 51/52 | 58/56 | 20 |
|  | 5220 | polyester-aluminium | aluminum1-transparentdiffuse1-aluminum1-open1-aluminum1-open1 | 60 | 4 | 52/56 | 54/60 | 20 |
|  | 6125 | polyolefin-aluminium | aluminum2-open1 | 126 | 4.6 | 61/- | 41/- | 25 |
|  | 6720 | polyolefin | whiteblack2-open1 | 120 | 4 | 67/68 | 70/71 | 20 |
| Tempa | 5155 | polyolefin-aluminium | aluminum1- transparentdiffuse1 | 136 | 4.6 | 51 | 58 | 55 |
|  | 5557 | polyester-aluminium | aluminum1-transparentdiffuse1 | 66 | 4 | 55/- | 61/- | 57 |
|  | 6360 | polyo.-alumin.-polye.-modacryl | aluminum2-transparent1-aluminum1-transparent1 | 121 | 4 | 63/67 | 66/70 | 60 |
|  | 6562 | polyester-aluminium | aluminum2-transparentdiffuse1-aluminum2-transparentdiffuse1-aluminum1- transparentdiffuse1 | 70 | 4 | 65/- | 68/- | 62 |
|  | 6960 | polyolefin-aluminium | aluminum2-transparent1-aluminum1-transparent1 | 182 | 4.6 | 69/73 | 72/75 | 60 |
|  | 7567 | polyester-aluminium | aluminum3-transparentdiffuse1 | 75 | 4 | 75/- | 78/- | 67 |

**S1 Table B. Svensson insect screen properties, all available information taken from the product sheets provided by the manufacturer.**

| **Type** | **Type no.** | **Material** | **Weight  g m^-2^** | **Ventilation reduction %** | **Warp density  yarns cm^-1^** | **Weft density  yarns cm^-1^** | **Opening mm** | **Energy saving %** |
| --- | --- | --- | --- | --- | --- | --- | --- | --- |
| Insect control | 1515 | polyolefin | 130 | 45 | 31.5 | 33 | 0.15 x 0.15 | 45 |
|  | 1535 | polyolefin | 106 | 40 | 31.5 | 20 | 0.15 x 0.35 | 40 |
|  | 2777 | polyolefin | 138 | 32 | 20 | 10 | 0.27 x 0.77 | 25 |
|  | 4045 | polyolefin | 73 | 30 | 19 | 17 | 0.40 x 0.45 | - |
