# Supplementary material for: Light quality characterization under climate screens and shade nets for controlled-environment agriculture
Source: PLoS One. 2018 Jun 25;13(6):e0199628. doi: 10.1371/journal.pone.0199628 (PMC6016941; doi:10.1371/journal.pone.0199628)
Supplement: S2 Table — (DOCX) [file pone.0199628.s002.docx]

**S2 Table A. Mallas Textiles shade net properties, all available information taken from the product sheets from the material supplier. All the nets are made of high-density polyethylene (HDPE). SN = shade net, PSN = patterned shade net. kgf= kilogram-force, kgf cm^-1^ = kilogram-force per centimeter.**

| **Type** | **Type colour** | **Colour pattern** | **Nominal shading factor** | **Weight  g m^-2^** | **Break resistance  Kgf cm^-1^** | **Weft  Kgf cm^-2^** | **Tear resistance Kgf** |
| --- | --- | --- | --- | --- | --- | --- | --- |
| SN | negro | black | 95 | 325 | 12 | 38 | 18 |
|  | cafe | dark brown | 95 | 325 | 12 | 38 | 18 |
|  | cafebeige | dark brown/light brown | 95 | 325 | 12 | 38 | 18 |
|  | azulblanco | blue/white | 95 | 325 | 12 | 38 | 18 |
|  | negro | black | 90 | 280 | 12 | 17 | 17 |
|  | verde | green | 90 | 280 | 12 | 17 | 17 |
|  | ambar | amber | 90 | 280 | 12 | 17 | 17 |
|  | azul | blue | 90 | 280 | 12 | 17 | 17 |
|  | negro | black | 80 | 260 | 11 | 19 | 15 |
|  | verde | green | 80 | 260 | 11 | 19 | 15 |
|  | negro | black | 70 | 240 | 10 | 18 | 15 |
|  | verde | green | 70 | 240 | 10 | 18 | 15 |
|  | negro | black | 50 | 220 | 9 | 15 | 13 |
|  | bicolor | grey | 50 | 220 | 9 | 15 | 13 |
|  | negro | black | 35 | 205 | 8 | 14 | 12 |
|  | blanco | white | 35 | 205 | 8 | 14 | 12 |
| PSN | negro | black | 90 | 105 | 12 | 16 | 10 |
|  | verde | green | 90 | 105 | 12 | 16 | 10 |
|  | ambar | amber | 90 | 105 | 12 | 16 | 10 |
|  | azul | blue | 90 | 105 | 12 | 16 | 10 |
|  | negro | black | 80 | 75 | 10 | 15 | 9 |
|  | verde | green | 80 | 75 | 10 | 15 | 9 |
|  | negro | black | 70 | 55 | 6 | 14 | 8 |
|  | verde | green | 70 | 55 | 6 | 14 | 8 |
|  | negro | black | 50 | 35 | 5.9 | 12 | 2.4 |
|  | negro | black | 35 | 33 | 4.7 | 11 | 2.4 |

**S2 Table B. Mallas Textiles insect net properties, all available information taken from the product sheets from the material supplier. All the nets are made of high-density polyethylene (HDPE). AT = antitrip, AA = antiafidos, AI = antiinsect.** **Den. = Denier =mass in grams per 9000 meters of the fiber. Tenacity g/Den. = Gram force per denier. Obtm = opening between threads in microns.**

| **Type** | **Type colour** | **Colour pattern** | **Mesh size (threads cm^-2^)** | **Weight  gm^-2^** | **Den.** | **Tenacity  gDen.^-1^** | **Obtm** |
| --- | --- | --- | --- | --- | --- | --- | --- |
| AT | cristal | lightgrey | 24x12 | 180 | 360 | 4.5 | 400 |
|  | cristal | lightgrey | 22x12 | 170 | 360 | 4.5 | 200 |
| AA | cristal | lightgrey | 20x10 | 150 | 360 | 4.5 | 300 |
|  | bicolor | darkgrey | 20x10 | 150 | 360 | 4.5 | 300 |
|  | negro | black | 16x16 | 140 | 360 | 4.5 | 250 |
|  | ambar | amber | 16x16 | 140 | 360 | 4.5 | 250 |
|  | negro | black | 16x10 | 130 | 360 | 4.5 | 500 |
|  | ambar | amber | 16x10 | 130 | 360 | 4.5 | 500 |
|  | cristal | lightgrey | 16x10 | 130 | 360 | 4.5 | 500 |
|  | bicolor | darkgrey | 16x10 | 130 | 360 | 4.5 | 500 |
| AI | cristal | lightgrey | 10x10 | 100 | 360 | 4.5 | 750 |
|  | bicolor | darkgrey | 10x10 | 100 | 360 | 4.5 | 750 |
