# Supplementary material for: Light quality characterization under climate screens and shade nets for controlled-environment agriculture
Source: PLoS One. 2018 Jun 25;13(6):e0199628. doi: 10.1371/journal.pone.0199628 (PMC6016941; doi:10.1371/journal.pone.0199628)
Supplement: S3 Table — (DOCX) [file pone.0199628.s003.docx]

**S3 Table. Spectral photon irradiance (µmol m^-2^ s^-1^) and percentage of total photon irradiance (280-900 nm) in each waveband calculated from simulated irradiances for the measurement dates in respective locations and local time in North Carolina. For the simulations, water vapour data was obtained from AERONET (Aerosol Robotic Network), providing continuous cloud-screened observations of precipitable water (online resource: http://aeronet.gsfc.nasa.gov, data were downloaded on 8 May 2018). Ozone column data was obtained from EOS Aura OMI OMUVB (Collection 3, PGE v1.3; for ascending orbit only with SZA < 88, online resource: https://avdc.gsfc.nasa.gov/pub/data/satellite/Aura/OMI/V03/L2OVP/OMUVB/, data were downloaded on 8 May 2018).**

|  | **UVB 280-315 nm** | | | | | | | | **UVA 315-400 nm** | | | | | | | |
| --- | --- | --- | --- | --- | --- | --- | --- | --- | --- | --- | --- | --- | --- | --- | --- | --- |
|  | **µmol m^-2^ s^-1^** | | | | **%** | | | | **µmol m^-2^ s^-1^** | | | | **%** | | | |
| **time** | **8.3.** | **4.4.** | **31.7.** | **10.8.** | **8.3.** | **4.4.** | **31.7.** | **10.8.** | **8.3.** | **4.4.** | **31.7.** | **10.8.** | **8.3.** | **4.4.** | **31.7.** | **10.8.** |
| 10:00 | 2.19 | 1.87 | 2.42 | 2.43 | 0.097 | 0.087 | 0.099 | 0.103 | 104.3 | 100.3 | 117.5 | 114.1 | 4.62 | 4.66 | 4.81 | 4.82 |
| 10:15 | 2.49 | 2.21 | 2.77 | 2.80 | 0.104 | 0.095 | 0.107 | 0.111 | 111.7 | 109.3 | 126.1 | 122.8 | 4.67 | 4.72 | 4.86 | 4.87 |
| 10:30 | 2.78 | 2.55 | 3.12 | 3.16 | 0.111 | 0.103 | 0.114 | 0.119 | 118.6 | 117.8 | 134.2 | 131.1 | 4.72 | 4.78 | 4.90 | 4.92 |
| 10:45 | 3.05 | 2.88 | 3.47 | 3.52 | 0.116 | 0.111 | 0.121 | 0.126 | 124.7 | 125.8 | 141.9 | 138.8 | 4.76 | 4.83 | 4.94 | 4.96 |
| 11:00 | 3.30 | 3.21 | 3.80 | 3.87 | 0.122 | 0.117 | 0.127 | 0.132 | 130.2 | 133.2 | 149.0 | 146.0 | 4.79 | 4.87 | 4.98 | 5.00 |
| 11:15 | 3.53 | 3.52 | 4.12 | 4.19 | 0.126 | 0.124 | 0.133 | 0.138 | 134.9 | 139.9 | 155.5 | 152.5 | 4.81 | 4.91 | 5.01 | 5.03 |
| 11:30 | 3.72 | 3.82 | 4.42 | 4.50 | 0.130 | 0.129 | 0.138 | 0.144 | 138.8 | 146.0 | 161.4 | 158.4 | 4.84 | 4.94 | 5.04 | 5.05 |
| 11:45 | 3.87 | 4.08 | 4.68 | 4.78 | 0.133 | 0.134 | 0.142 | 0.148 | 141.9 | 151.4 | 166.6 | 163.7 | 4.85 | 4.97 | 5.06 | 5.08 |
| 12:00 | 3.99 | 4.32 | 4.92 | 5.02 | 0.135 | 0.138 | 0.146 | 0.152 | 144.2 | 156.1 | 171.1 | 168.2 | 4.87 | 4.99 | 5.08 | 5.10 |
| 12:15 | 4.06 | 4.51 | 5.12 | 5.23 | 0.136 | 0.141 | 0.149 | 0.155 | 145.7 | 159.9 | 174.8 | 172.0 | 4.87 | 5.00 | 5.09 | 5.11 |
| 12:30 | 4.10 | 4.67 | 5.29 | 5.40 | 0.137 | 0.144 | 0.152 | 0.158 | 146.3 | 163.0 | 177.9 | 175.0 | 4.88 | 5.02 | 5.10 | 5.12 |
| 12:45 | 4.08 | 4.79 | 5.41 | 5.53 | 0.136 | 0.146 | 0.154 | 0.160 | 146.0 | 165.2 | 180.1 | 177.2 | 4.87 | 5.03 | 5.11 | 5.13 |
| 13:00 | 4.03 | 4.87 | 5.50 | 5.61 | 0.135 | 0.147 | 0.155 | 0.161 | 144.9 | 166.6 | 181.6 | 178.7 | 4.87 | 5.03 | 5.12 | 5.14 |
| 13:15 | 3.93 | 4.90 | 5.54 | 5.65 | 0.134 | 0.148 | 0.155 | 0.162 | 143.0 | 167.2 | 182.3 | 179.3 | 4.86 | 5.04 | 5.12 | 5.14 |
| 13:30 | 3.79 | 4.89 | 5.53 | 5.64 | 0.131 | 0.147 | 0.155 | 0.162 | 140.3 | 166.9 | 182.2 | 179.2 | 4.84 | 5.03 | 5.12 | 5.14 |
| 13:45 | 3.62 | 4.83 | 5.48 | 5.58 | 0.128 | 0.146 | 0.155 | 0.161 | 136.7 | 165.8 | 181.4 | 178.3 | 4.83 | 5.03 | 5.12 | 5.13 |
| 14:00 | 3.41 | 4.72 | 5.39 | 5.49 | 0.124 | 0.145 | 0.153 | 0.159 | 132.3 | 163.9 | 179.7 | 176.5 | 4.80 | 5.02 | 5.11 | 5.13 |

Continues on the next page.

Continuing from the previous page.

|  | **B100 400-500 nm** | | | | | | | | **G100 500-600 nm** | | | | | | | |
| --- | --- | --- | --- | --- | --- | --- | --- | --- | --- | --- | --- | --- | --- | --- | --- | --- |
|  | **µmol m^-2^ s^-1^** | | | | **%** | | | | **µmol m^-2^ s^-1^** | | | | **%** | | | |
| **time** | **8.3.** | **4.4.** | **31.7.** | **10.8.** | **8.3.** | **4.4.** | **31.7.** | **10.8.** | **8.3.** | **4.4.** | **31.7.** | **10.8.** | **8.3.** | **4.4.** | **31.7.** | **10.8.** |
| 10:00 | 360.2 | 348.3 | 398.8 | 388.7 | 15.95 | 16.18 | 16.32 | 16.42 | 458.3 | 440.1 | 500.1 | 487.4 | 20.29 | 20.45 | 20.47 | 20.59 |
| 10:15 | 382.7 | 375.4 | 424.3 | 414.6 | 16.00 | 16.23 | 16.35 | 16.45 | 485.8 | 473.3 | 531.4 | 519.0 | 20.31 | 20.46 | 20.48 | 20.59 |
| 10:30 | 403.1 | 400.9 | 448.4 | 438.9 | 16.04 | 16.27 | 16.38 | 16.48 | 510.9 | 504.5 | 560.8 | 548.7 | 20.32 | 20.48 | 20.49 | 20.60 |
| 10:45 | 421.4 | 424.6 | 470.9 | 461.6 | 16.07 | 16.31 | 16.41 | 16.50 | 533.3 | 533.6 | 588.1 | 576.4 | 20.33 | 20.49 | 20.49 | 20.61 |
| 11:00 | 437.6 | 446.5 | 491.6 | 482.6 | 16.09 | 16.33 | 16.43 | 16.52 | 553.0 | 560.3 | 613.4 | 602.0 | 20.34 | 20.50 | 20.50 | 20.61 |
| 11:15 | 451.4 | 466.4 | 510.5 | 501.7 | 16.12 | 16.36 | 16.45 | 16.54 | 570.0 | 584.6 | 636.5 | 625.2 | 20.35 | 20.50 | 20.50 | 20.61 |
| 11:30 | 463.0 | 484.3 | 527.5 | 518.8 | 16.13 | 16.38 | 16.46 | 16.55 | 584.1 | 606.4 | 657.2 | 646.1 | 20.36 | 20.51 | 20.51 | 20.61 |
| 11:45 | 472.1 | 500.0 | 542.6 | 534.0 | 16.15 | 16.39 | 16.47 | 16.56 | 595.3 | 625.6 | 675.5 | 664.6 | 20.36 | 20.51 | 20.51 | 20.61 |
| 12:00 | 478.9 | 513.5 | 555.5 | 547.0 | 16.16 | 16.41 | 16.48 | 16.57 | 603.5 | 642.0 | 691.3 | 680.4 | 20.36 | 20.51 | 20.51 | 20.61 |
| 12:15 | 483.1 | 524.7 | 566.4 | 557.9 | 16.16 | 16.42 | 16.49 | 16.58 | 608.7 | 655.7 | 704.5 | 693.7 | 20.36 | 20.52 | 20.51 | 20.62 |
| 12:30 | 484.9 | 533.6 | 575.1 | 566.6 | 16.16 | 16.43 | 16.50 | 16.59 | 610.9 | 666.5 | 715.1 | 704.3 | 20.36 | 20.52 | 20.51 | 20.62 |
| 12:45 | 484.2 | 540.0 | 581.6 | 573.1 | 16.16 | 16.43 | 16.50 | 16.59 | 610.1 | 674.4 | 723.0 | 712.1 | 20.36 | 20.52 | 20.52 | 20.62 |
| 13:00 | 481.0 | 544.1 | 585.9 | 577.2 | 16.16 | 16.44 | 16.51 | 16.59 | 606.2 | 679.3 | 728.2 | 717.2 | 20.36 | 20.52 | 20.52 | 20.62 |
| 13:15 | 475.4 | 545.8 | 587.9 | 579.2 | 16.15 | 16.44 | 16.51 | 16.59 | 599.2 | 681.4 | 730.7 | 719.5 | 20.36 | 20.52 | 20.52 | 20.62 |
| 13:30 | 467.3 | 545.0 | 587.7 | 578.8 | 16.14 | 16.44 | 16.51 | 16.59 | 589.4 | 680.4 | 730.4 | 719.1 | 20.36 | 20.52 | 20.52 | 20.62 |
| 13:45 | 456.7 | 541.8 | 585.2 | 576.1 | 16.12 | 16.43 | 16.51 | 16.59 | 576.5 | 676.5 | 727.4 | 715.8 | 20.35 | 20.52 | 20.52 | 20.62 |
| 14:00 | 443.9 | 536.2 | 580.5 | 571.1 | 16.10 | 16.43 | 16.50 | 16.59 | 560.8 | 669.7 | 721.6 | 709.7 | 20.35 | 20.52 | 20.52 | 20.62 |

Continues on the next page.

Continuing from the previous page.

|  | **R100 600-700 nm** | | | | | | | | **FR100 700-800 nm** | | | | | | | |
| --- | --- | --- | --- | --- | --- | --- | --- | --- | --- | --- | --- | --- | --- | --- | --- | --- |
|  | **µmol m^-2^ s^-1^** | | | | **%** | | | | **µmol m^-2^ s^-1^** | | | | **%** | | | |
| **time** | **8.3.** | **4.4.** | **31.7.** | **10.8.** | **8.3.** | **4.4.** | **31.7.** | **10.8.** | **8.3.** | **4.4.** | **31.7.** | **10.8.** | **8.3.** | **4.4.** | **31.7.** | **10.8.** |
| 10:00 | 478.4 | 459.6 | 519.8 | 506.5 | 21.18 | 21.35 | 21.27 | 21.39 | 443.7 | 416.8 | 470.9 | 452.2 | 19.64 | 19.36 | 19.27 | 19.10 |
| 10:15 | 506.1 | 493.1 | 551.3 | 538.4 | 21.16 | 21.32 | 21.25 | 21.36 | 468.9 | 446.9 | 499.2 | 480.6 | 19.60 | 19.32 | 19.24 | 19.07 |
| 10:30 | 531.4 | 524.5 | 580.9 | 568.3 | 21.14 | 21.29 | 21.22 | 21.33 | 491.7 | 475.1 | 525.9 | 507.4 | 19.56 | 19.29 | 19.21 | 19.05 |
| 10:45 | 554.0 | 553.8 | 608.4 | 596.1 | 21.12 | 21.27 | 21.20 | 21.31 | 512.2 | 501.5 | 550.7 | 532.4 | 19.53 | 19.26 | 19.19 | 19.03 |
| 11:00 | 573.8 | 580.7 | 633.9 | 621.8 | 21.11 | 21.24 | 21.18 | 21.29 | 530.3 | 525.7 | 573.7 | 555.4 | 19.51 | 19.23 | 19.17 | 19.01 |
| 11:15 | 590.9 | 605.2 | 657.0 | 645.2 | 21.10 | 21.22 | 21.17 | 21.27 | 545.8 | 547.8 | 594.6 | 576.4 | 19.49 | 19.21 | 19.16 | 19.00 |
| 11:30 | 605.1 | 627.1 | 677.9 | 666.3 | 21.09 | 21.21 | 21.15 | 21.25 | 558.7 | 567.6 | 613.4 | 595.3 | 19.47 | 19.19 | 19.14 | 18.99 |
| 11:45 | 616.4 | 646.4 | 696.3 | 684.8 | 21.08 | 21.19 | 21.14 | 21.24 | 568.9 | 585.0 | 630.1 | 611.9 | 19.46 | 19.18 | 19.13 | 18.98 |
| 12:00 | 624.6 | 662.9 | 712.2 | 700.8 | 21.07 | 21.18 | 21.13 | 21.23 | 576.4 | 599.9 | 644.4 | 626.3 | 19.45 | 19.17 | 19.12 | 18.97 |
| 12:15 | 629.9 | 676.6 | 725.5 | 714.1 | 21.07 | 21.17 | 21.12 | 21.22 | 581.2 | 612.3 | 656.4 | 638.3 | 19.44 | 19.16 | 19.11 | 18.97 |
| 12:30 | 632.1 | 687.5 | 736.1 | 724.7 | 21.07 | 21.17 | 21.12 | 21.21 | 583.2 | 622.1 | 666.1 | 647.8 | 19.44 | 19.15 | 19.11 | 18.96 |
| 12:45 | 631.2 | 695.5 | 744.1 | 732.6 | 21.07 | 21.16 | 21.11 | 21.21 | 582.4 | 629.3 | 673.3 | 654.9 | 19.44 | 19.15 | 19.10 | 18.96 |
| 13:00 | 627.3 | 700.5 | 749.3 | 737.7 | 21.07 | 21.16 | 21.11 | 21.20 | 578.8 | 633.8 | 678.0 | 659.5 | 19.45 | 19.15 | 19.10 | 18.96 |
| 13:15 | 620.3 | 702.5 | 751.8 | 740.1 | 21.08 | 21.16 | 21.11 | 21.20 | 572.5 | 635.7 | 680.3 | 661.6 | 19.45 | 19.14 | 19.10 | 18.96 |
| 13:30 | 610.4 | 701.5 | 751.5 | 739.6 | 21.08 | 21.16 | 21.11 | 21.20 | 563.5 | 634.8 | 680.0 | 661.2 | 19.46 | 19.14 | 19.10 | 18.96 |
| 13:45 | 597.4 | 697.6 | 748.5 | 736.3 | 21.09 | 21.16 | 21.11 | 21.21 | 551.7 | 631.3 | 677.3 | 658.2 | 19.48 | 19.15 | 19.10 | 18.96 |
| 14:00 | 581.6 | 690.8 | 742.7 | 730.2 | 21.10 | 21.16 | 21.11 | 21.21 | 537.3 | 625.1 | 672.0 | 652.8 | 19.50 | 19.15 | 19.11 | 18.96 |

Continues on the next page.

Continuing from the previous page.

|  | **NIR100 800-900 nm** | | | | | | | |
| --- | --- | --- | --- | --- | --- | --- | --- | --- |
|  | **µmol m^-2^ s^-1^** | | | | **%** | | | |
| **time** | **8.3.** | **4.4.** | **31.7.** | **10.8.** | **8.3.** | **4.4.** | **31.7.** | **10.8.** |
| 10:00 | 411.8 | 385.4 | 434.1 | 416.3 | 18.23 | 17.91 | 17.77 | 17.58 |
| 10:15 | 434.6 | 412.6 | 459.7 | 441.9 | 18.17 | 17.84 | 17.72 | 17.54 |
| 10:30 | 455.4 | 438.2 | 483.7 | 466.1 | 18.12 | 17.79 | 17.67 | 17.50 |
| 10:45 | 474.0 | 462.0 | 506.2 | 488.6 | 18.07 | 17.74 | 17.64 | 17.46 |
| 11:00 | 490.4 | 483.9 | 526.9 | 509.3 | 18.04 | 17.70 | 17.61 | 17.44 |
| 11:15 | 504.5 | 503.8 | 545.7 | 528.2 | 18.01 | 17.67 | 17.58 | 17.41 |
| 11:30 | 516.2 | 521.6 | 562.7 | 545.2 | 17.99 | 17.64 | 17.56 | 17.39 |
| 11:45 | 525.5 | 537.3 | 577.7 | 560.2 | 17.97 | 17.62 | 17.54 | 17.38 |
| 12:00 | 532.3 | 550.8 | 590.6 | 573.1 | 17.96 | 17.60 | 17.53 | 17.36 |
| 12:15 | 536.6 | 562.0 | 601.5 | 583.9 | 17.95 | 17.59 | 17.51 | 17.35 |
| 12:30 | 538.4 | 570.8 | 610.1 | 592.5 | 17.95 | 17.57 | 17.50 | 17.34 |
| 12:45 | 537.7 | 577.3 | 616.6 | 598.9 | 17.95 | 17.57 | 17.50 | 17.34 |
| 13:00 | 534.5 | 581.4 | 620.9 | 603.0 | 17.96 | 17.56 | 17.49 | 17.33 |
| 13:15 | 528.8 | 583.1 | 622.9 | 604.9 | 17.97 | 17.56 | 17.49 | 17.33 |
| 13:30 | 520.5 | 582.3 | 622.7 | 604.5 | 17.98 | 17.56 | 17.49 | 17.33 |
| 13:45 | 509.9 | 579.1 | 620.2 | 601.9 | 18.00 | 17.56 | 17.49 | 17.33 |
| 14:00 | 496.8 | 573.5 | 615.5 | 596.9 | 18.03 | 17.57 | 17.50 | 17.34 |
