# Supplementary material for: Light quality characterization under climate screens and shade nets for controlled-environment agriculture
Source: PLoS One. 2018 Jun 25;13(6):e0199628. doi: 10.1371/journal.pone.0199628 (PMC6016941; doi:10.1371/journal.pone.0199628)
Supplement: S1 Fig — (PDF) [file pone.0199628.s004.pdf]

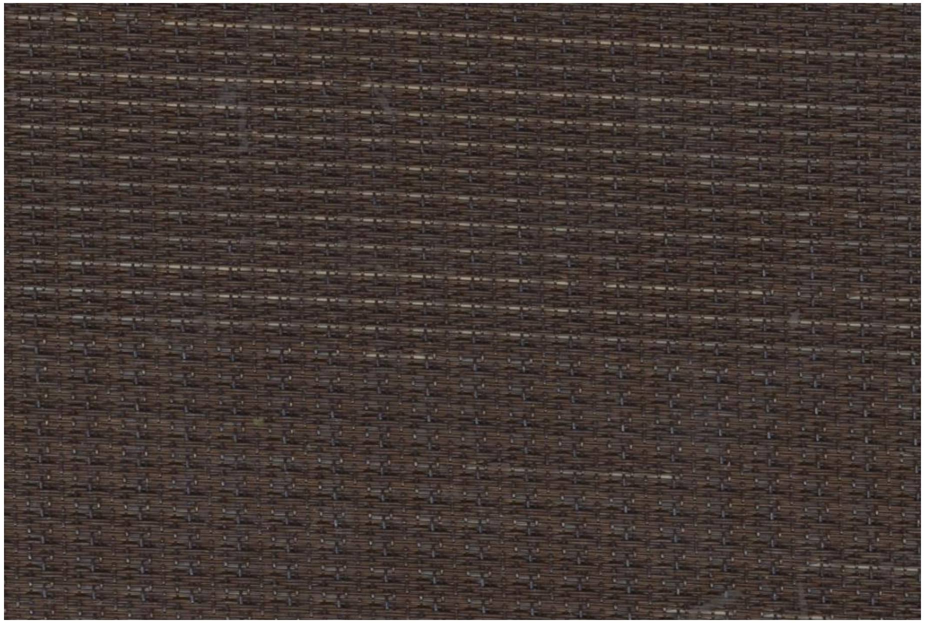
Mallas Textiles Sombra 95 dark brown

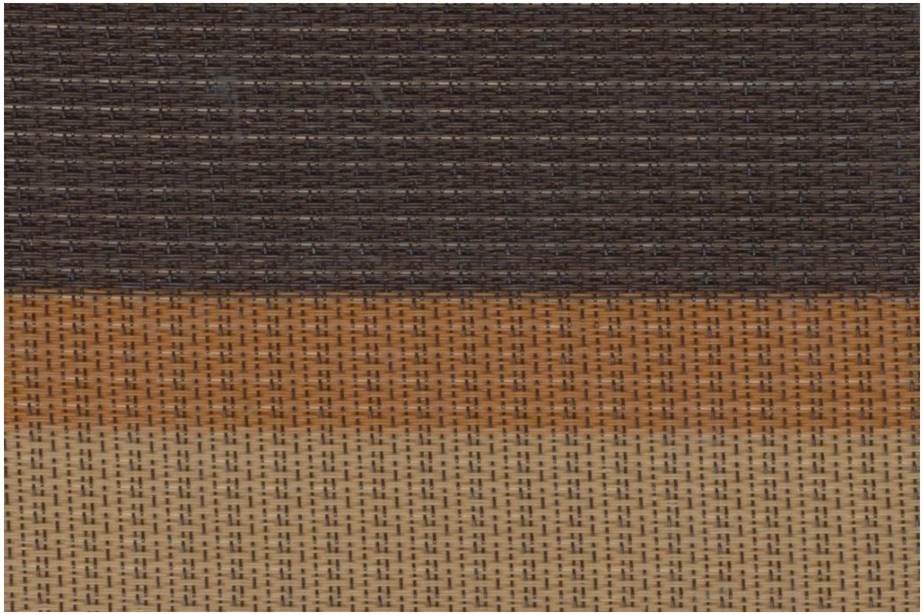
Mallas Textiles Sombra 95 dark brown/light brown

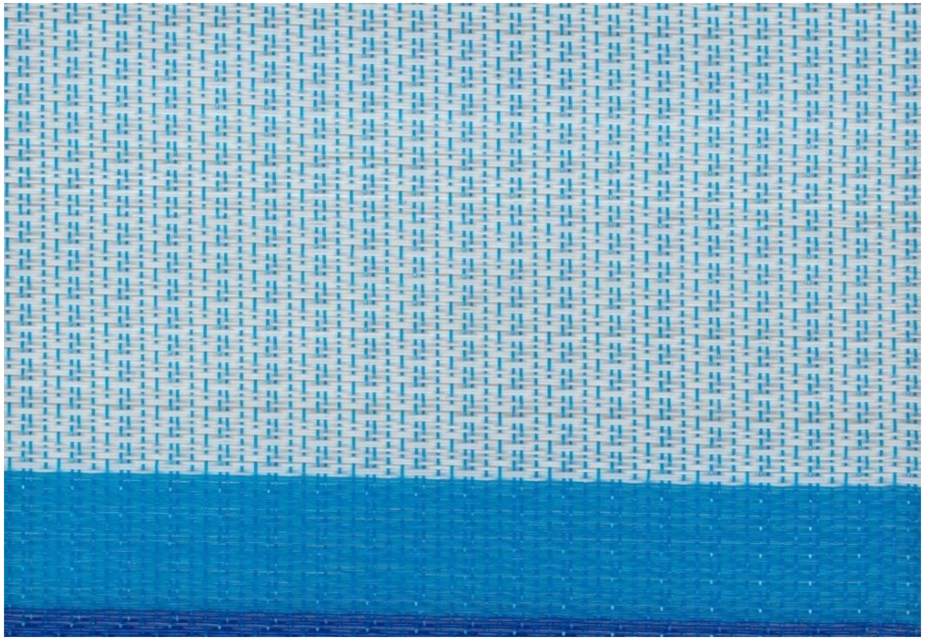
Mallas Textiles Sombra 95 blue/white

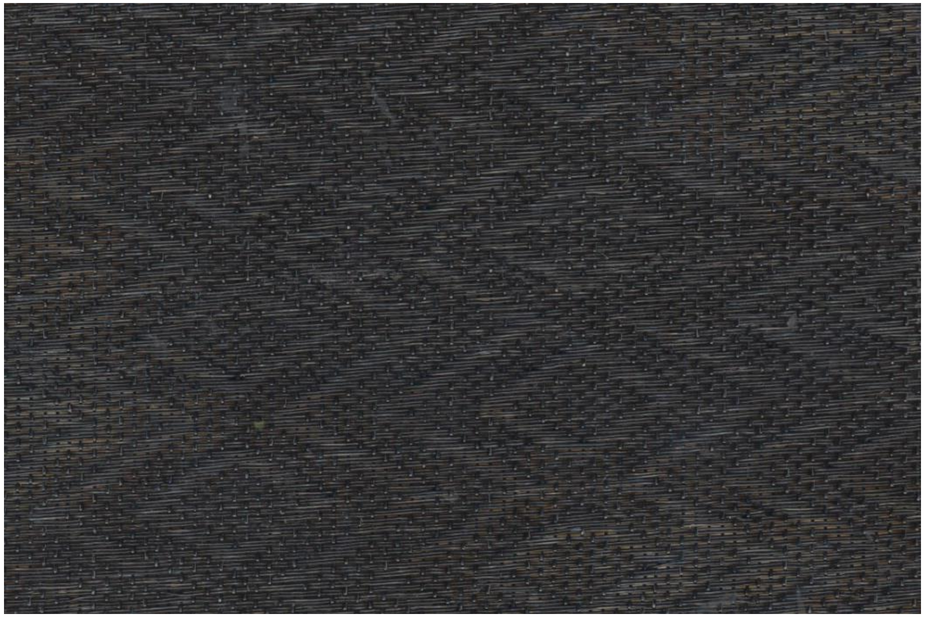
Mallas Textiles Sombra 90 black

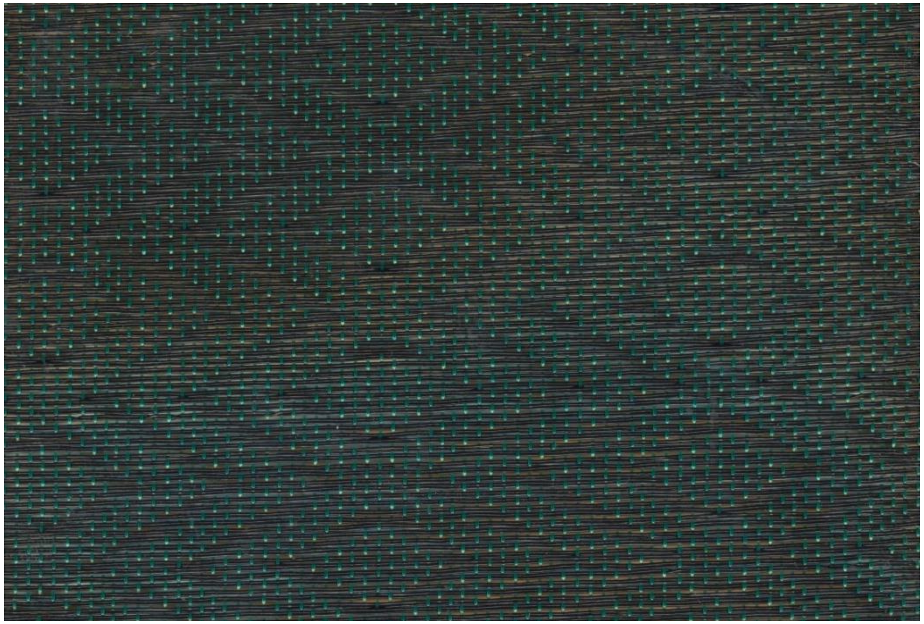
Mallas Textiles Sombra 90 green

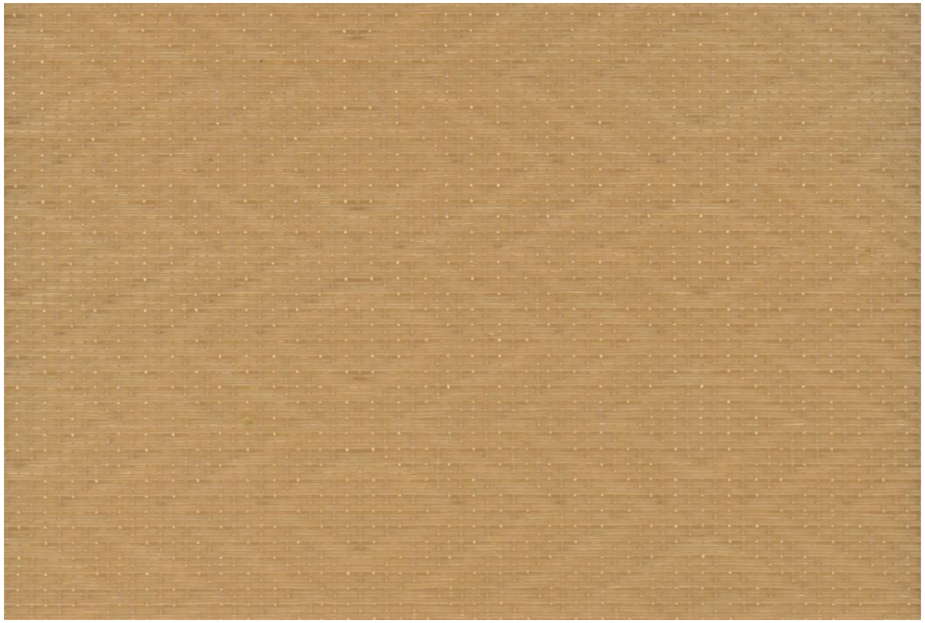
Mallas Textiles Sombra 90 amber

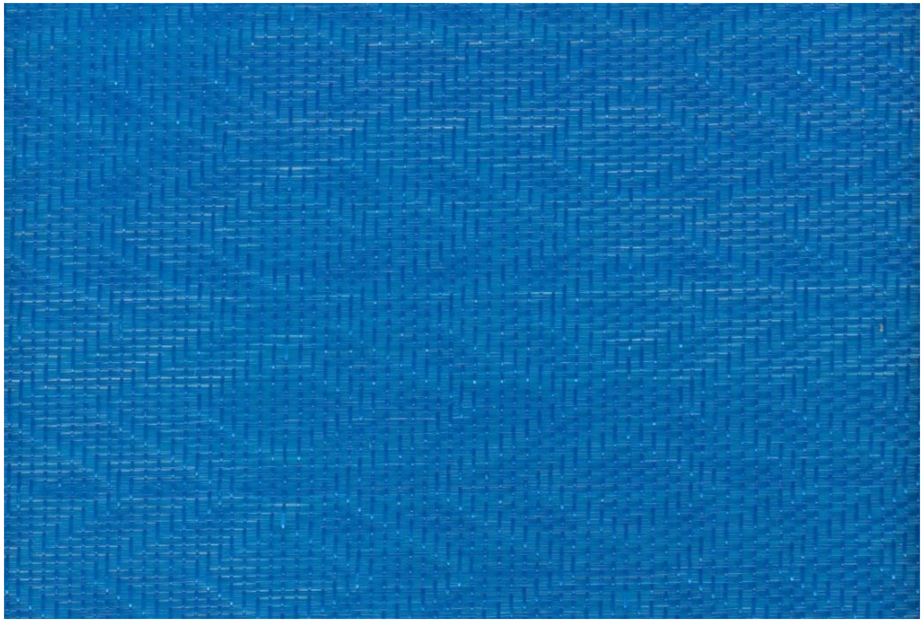
Mallas Textiles Sombra 90 blue

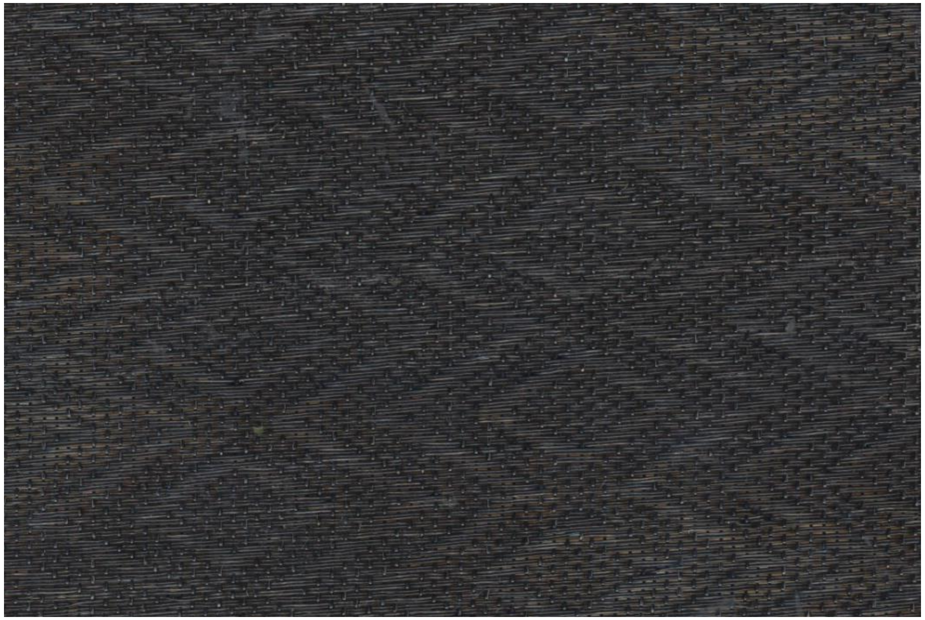
Mallas Textiles Sombra 80 black

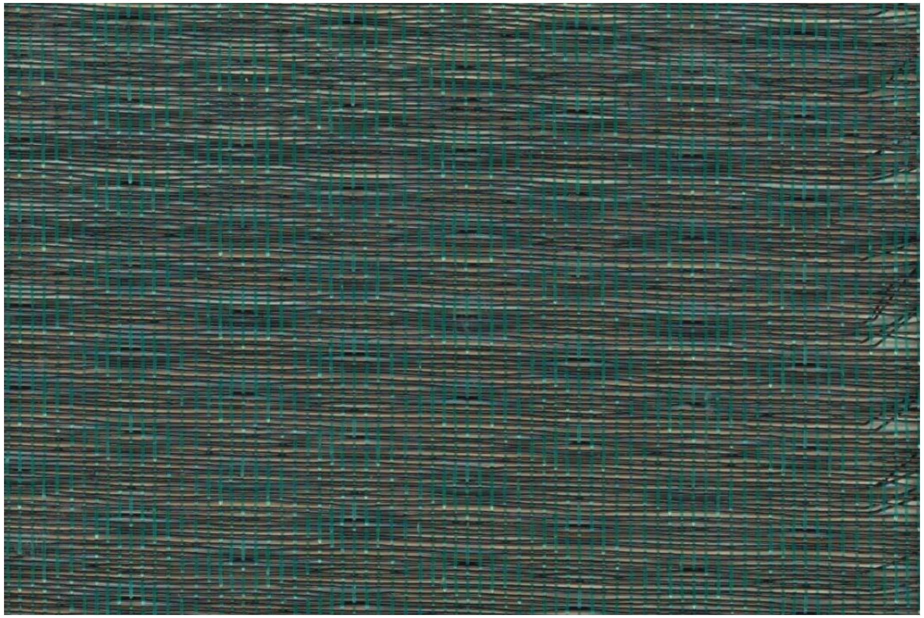
Mallas Textiles Sombra 80 green

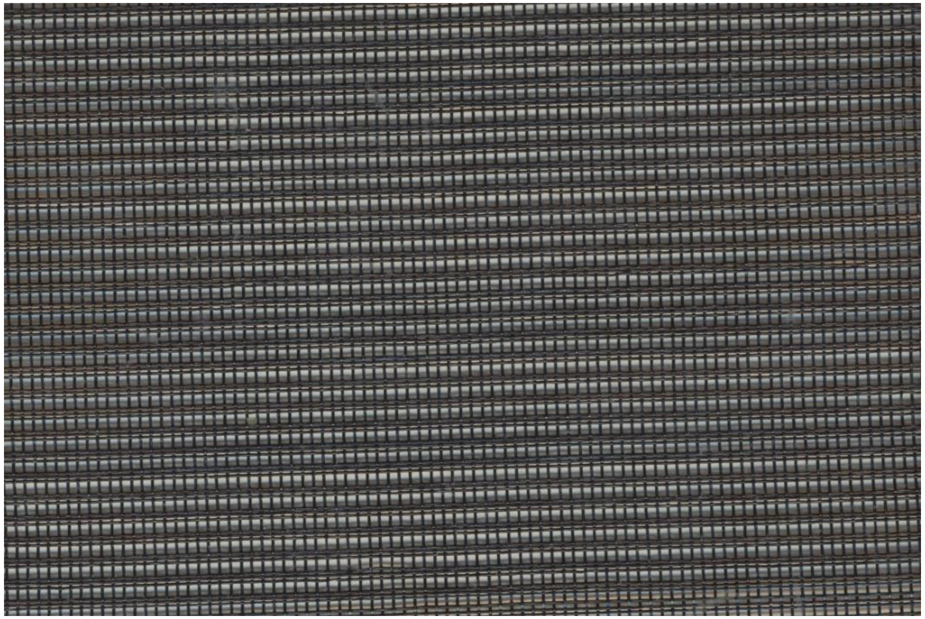
Mallas Textiles Sombra 70 black

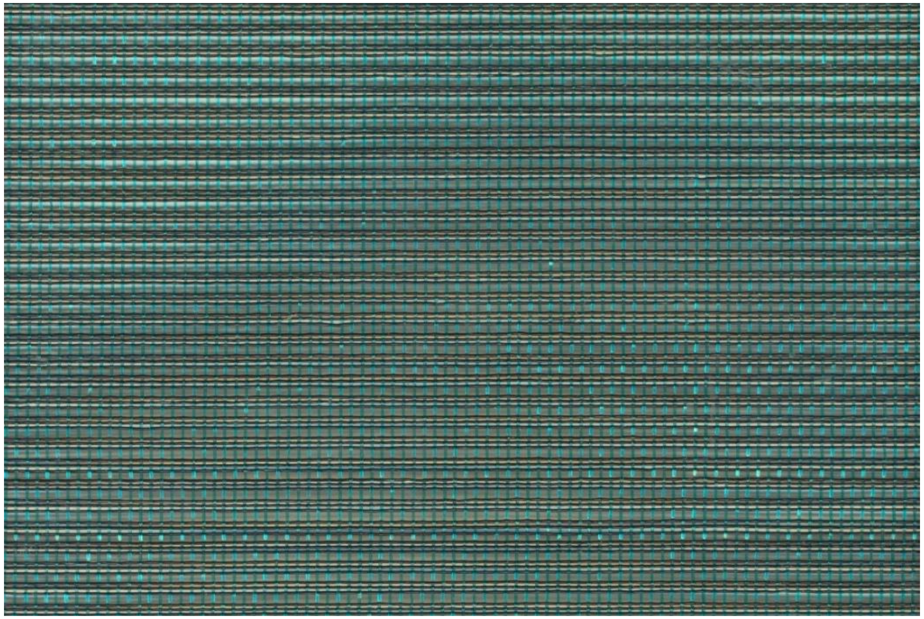
Mallas Textiles Sombra 70 green

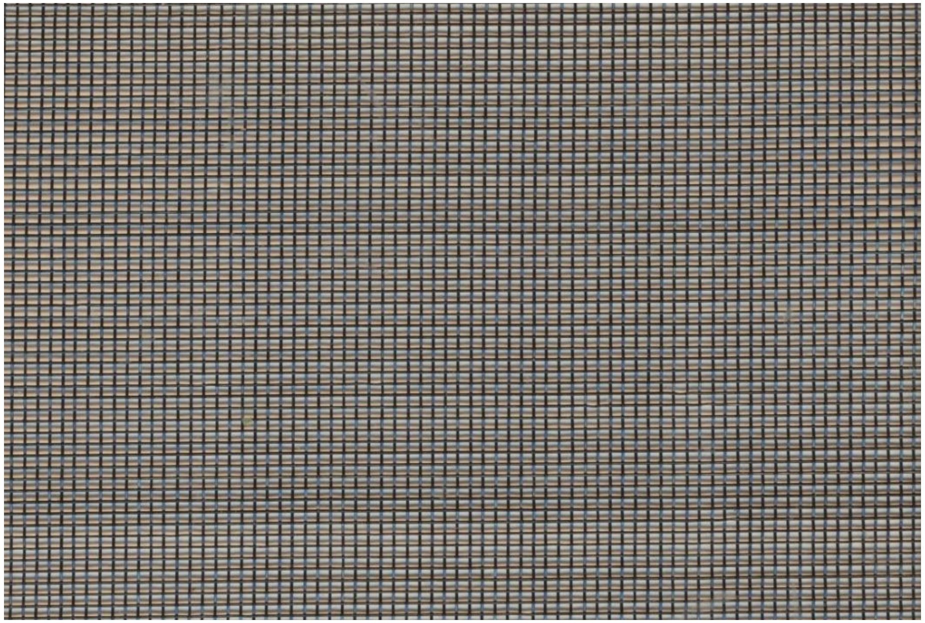

Mallas Textiles Sombra 50 black

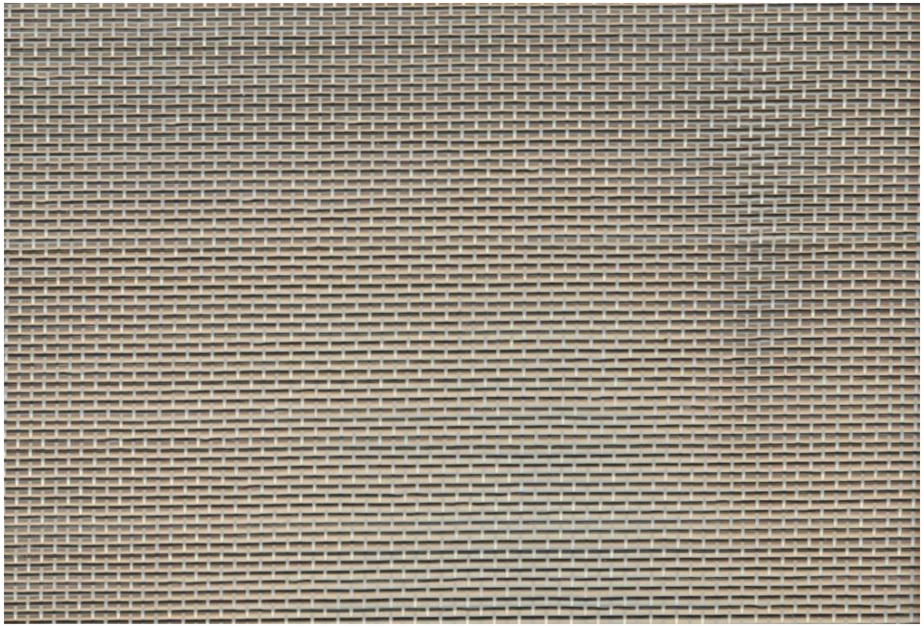

Mallas Textiles Sombra 50 darkgrey

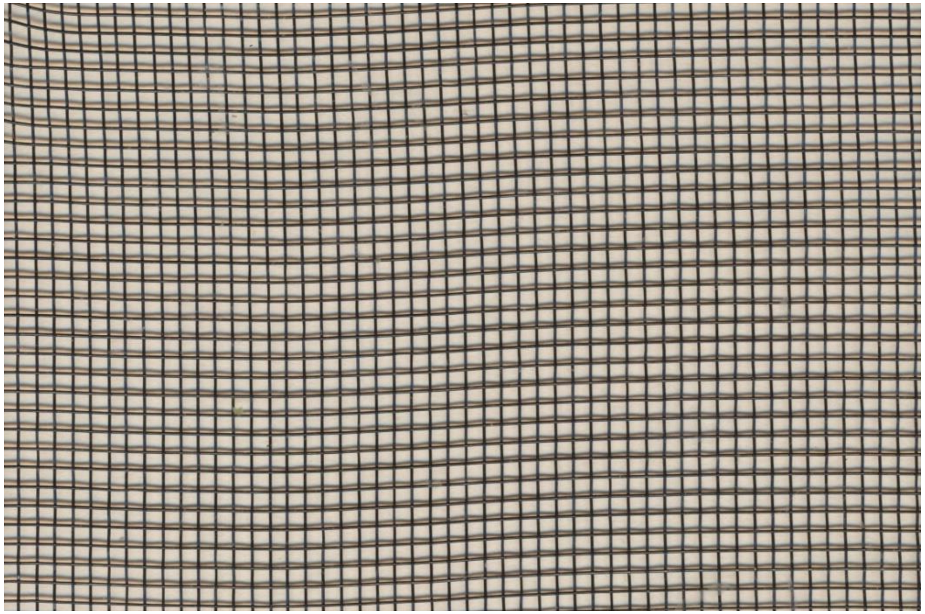

Mallas Textiles Sombra 35 black

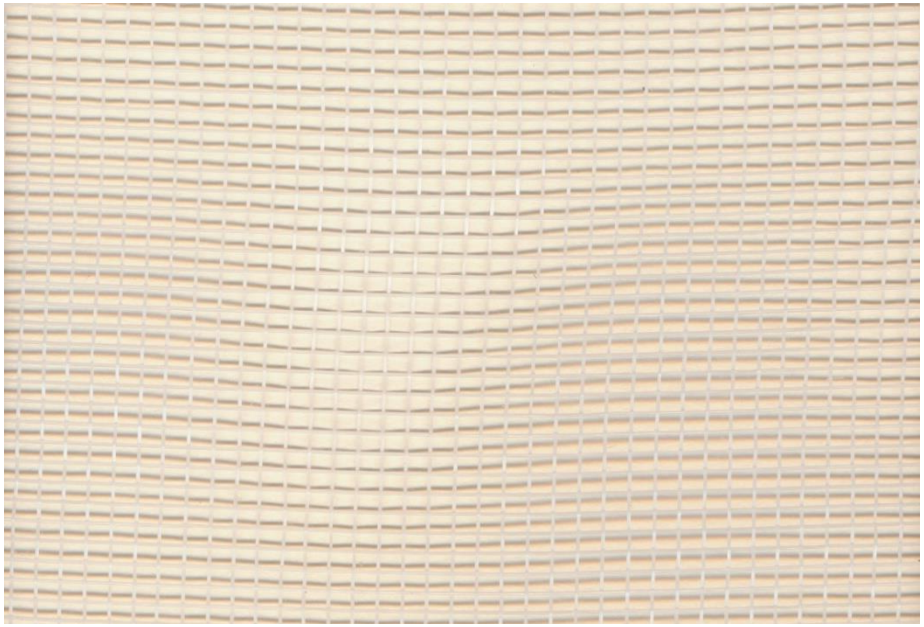

Mallas Textiles Sombra 35 white

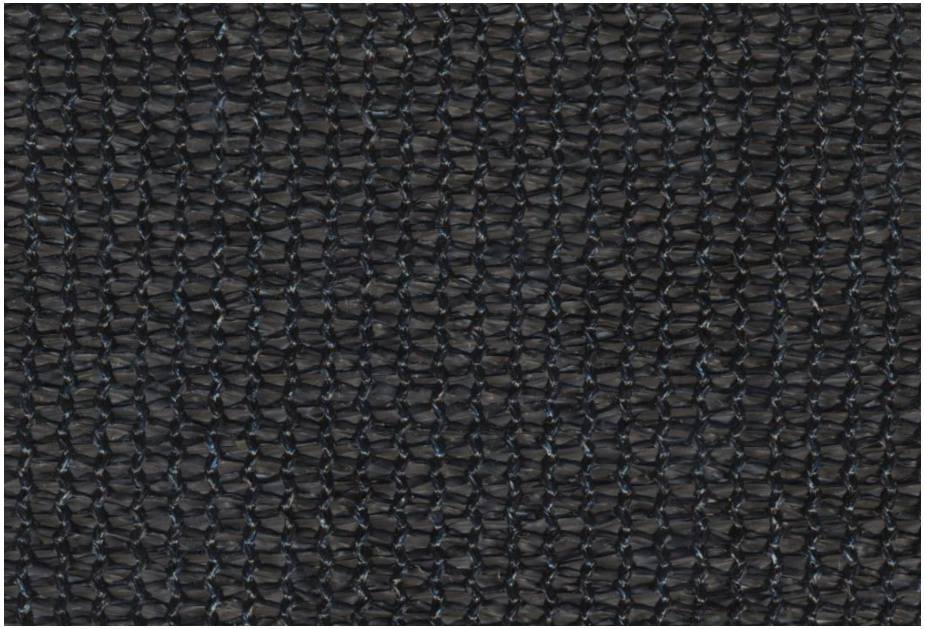

Mallas Textiles Sombra raschel 90 black

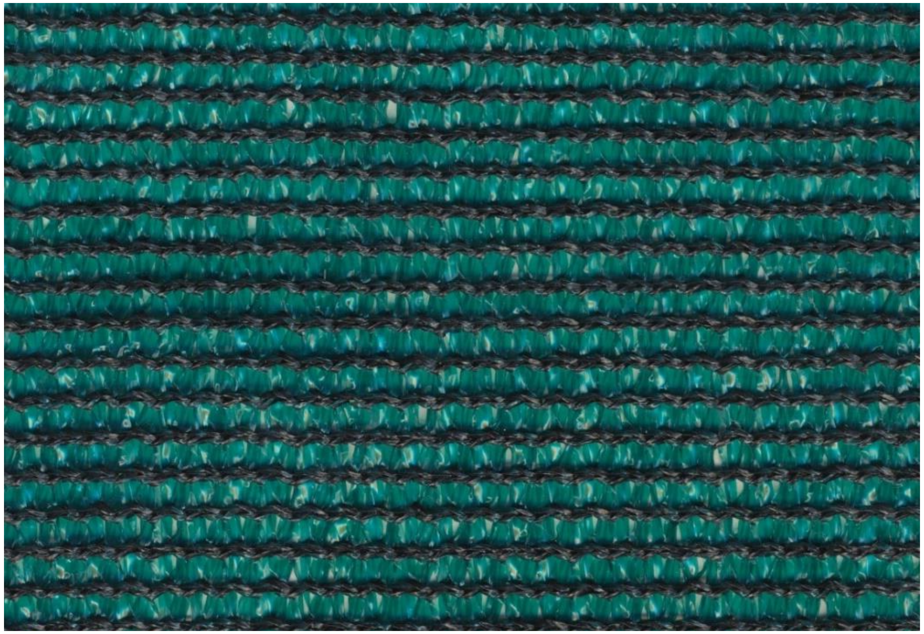

Mallas Textiles Sombra raschel 90 green

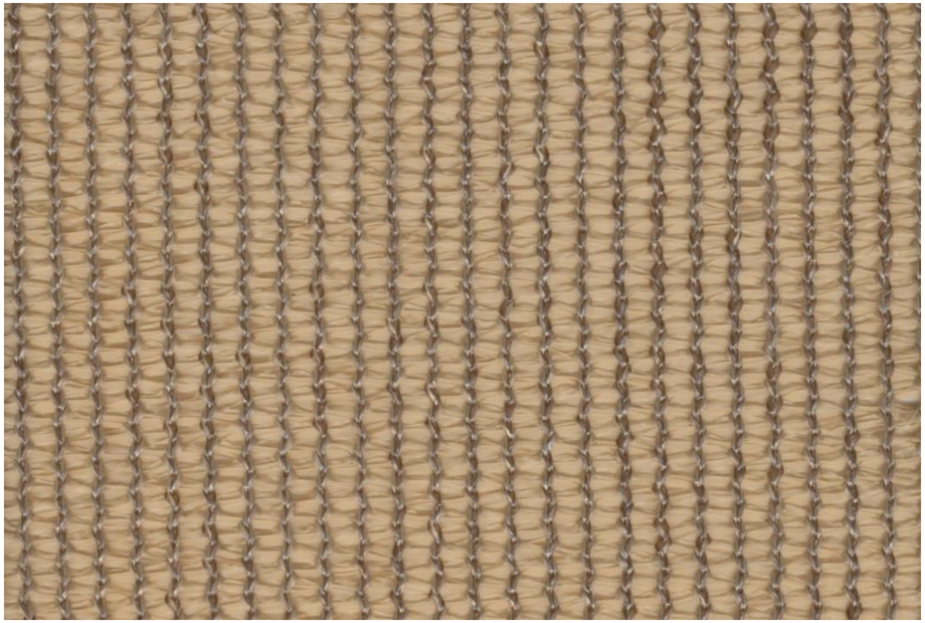

Mallas Textiles Sombra raschel 90 amber

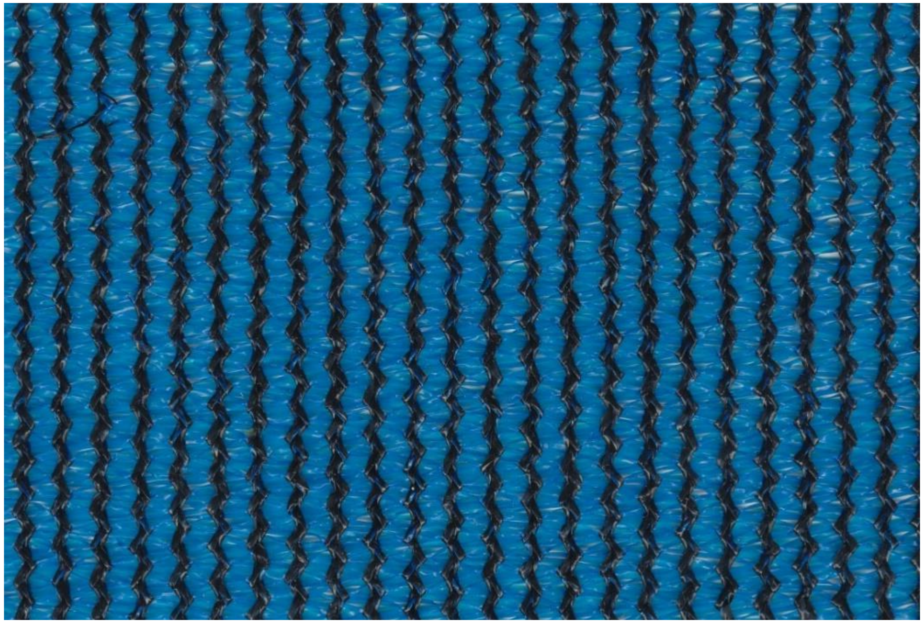

Mallas Textiles Sombra raschel 90 blue

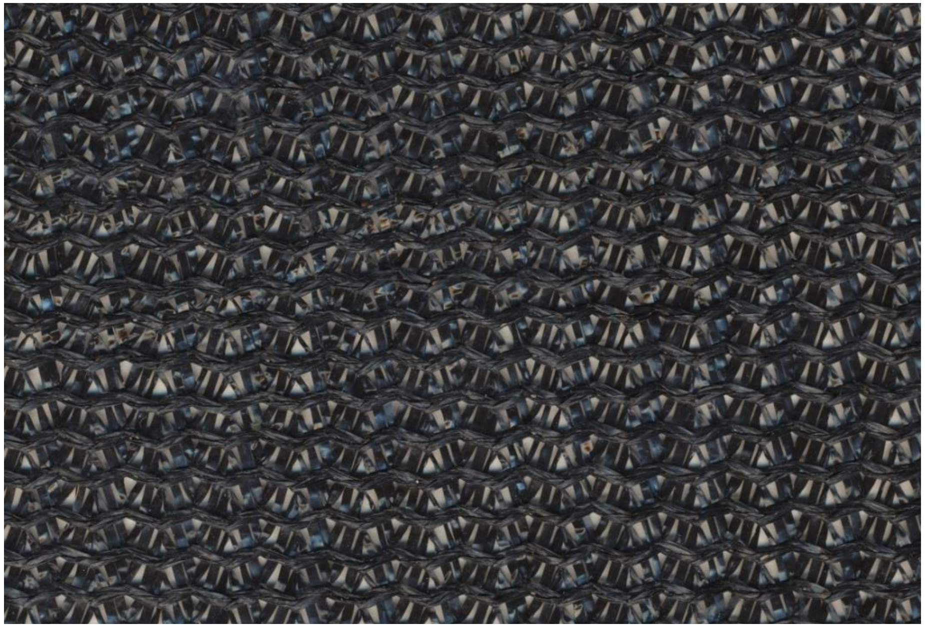
Mallas Textiles Sombra raschel 80 black

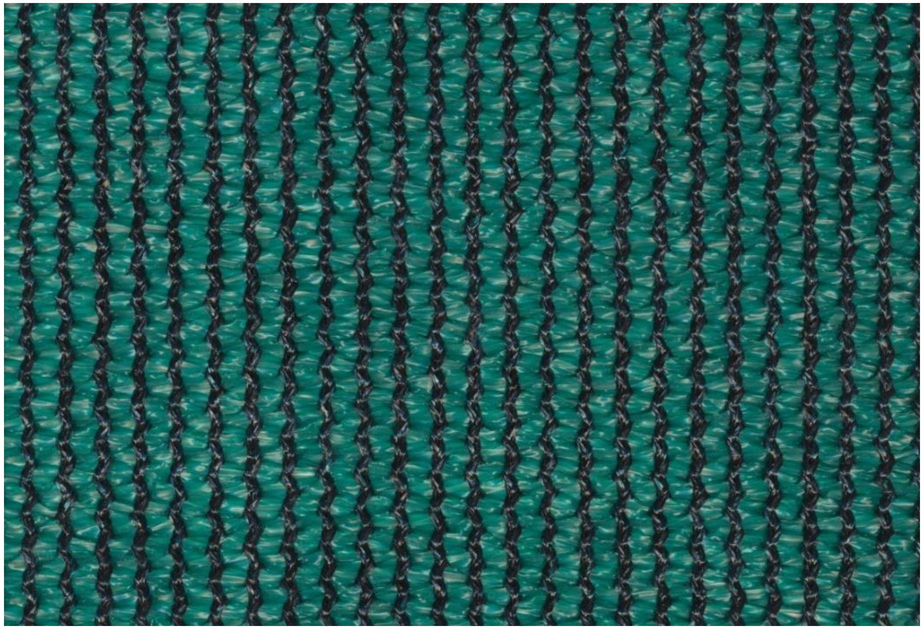
Mallas Textiles Sombra raschel 80 green

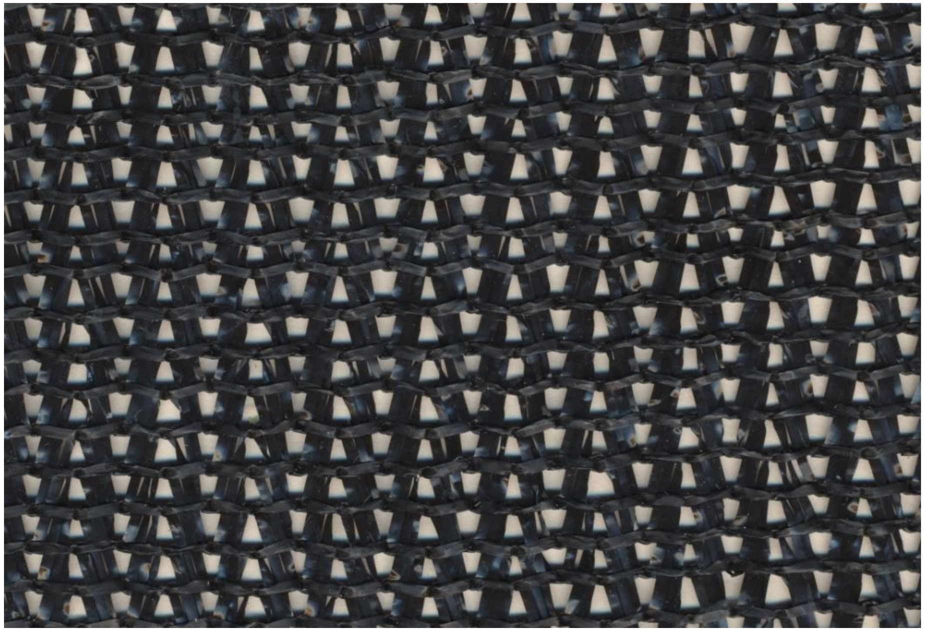
Mallas Textiles Sombra raschel 70 black

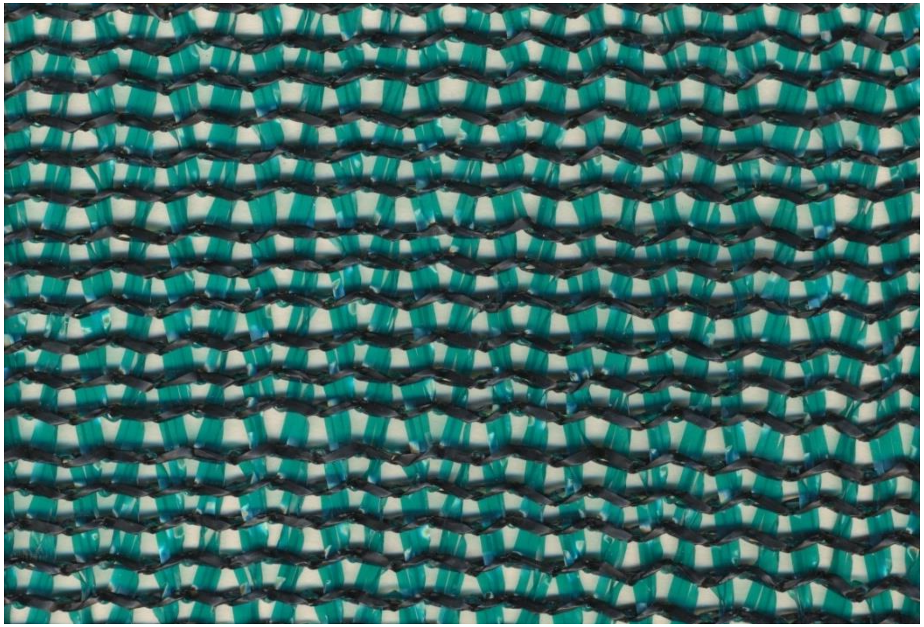
Mallas Textiles Sombra raschel 70 green

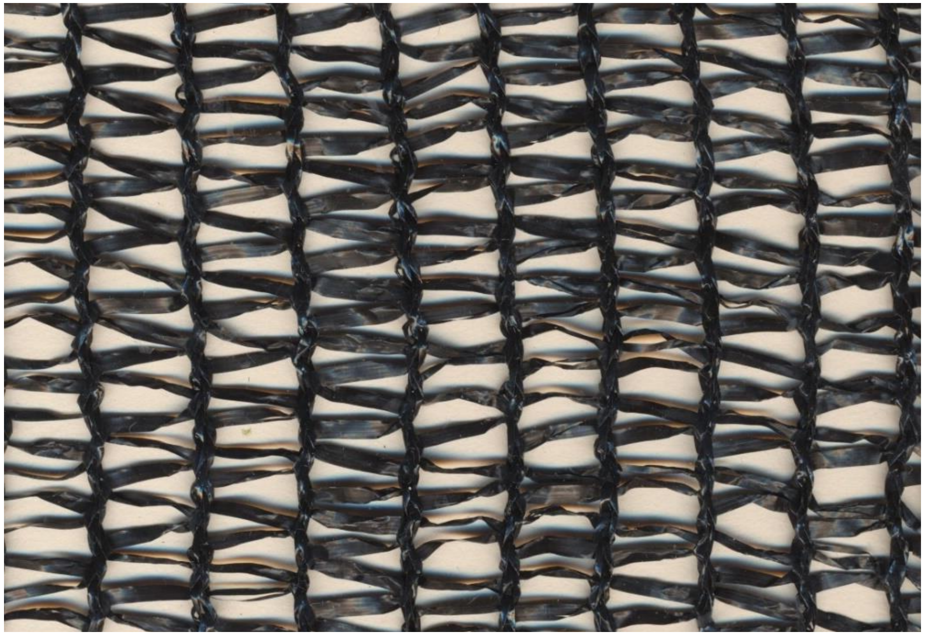

Mallas Textiles Sombra raschel 50 black

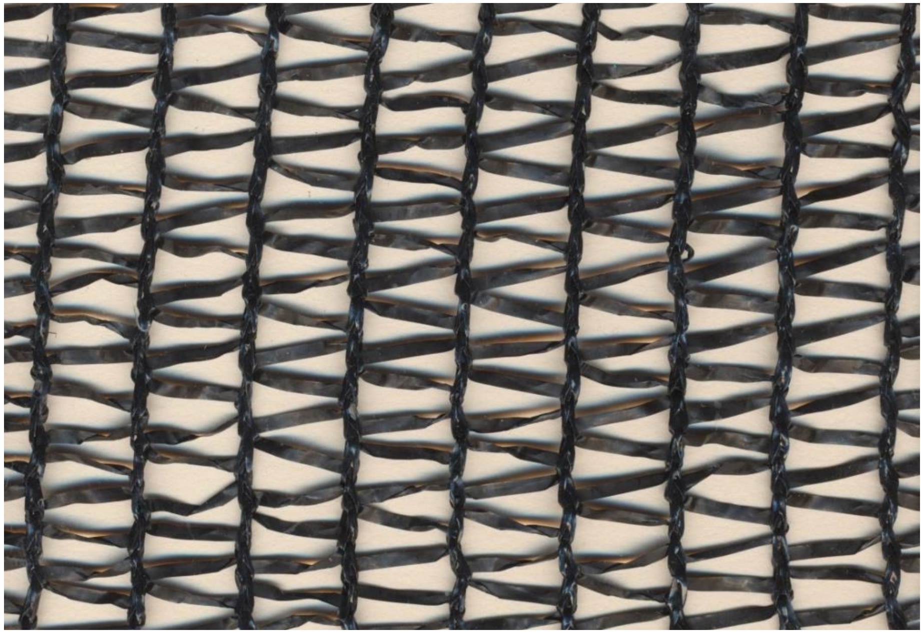

Mallas Textiles Sombra raschel 35 black

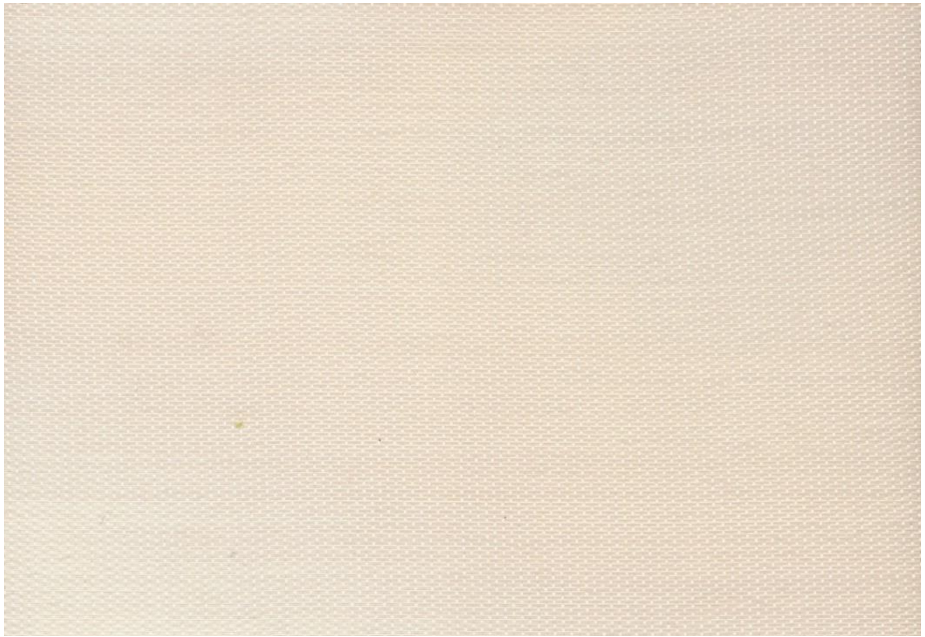

Mallastextiles Anti-Trip 60x30 lightgrey

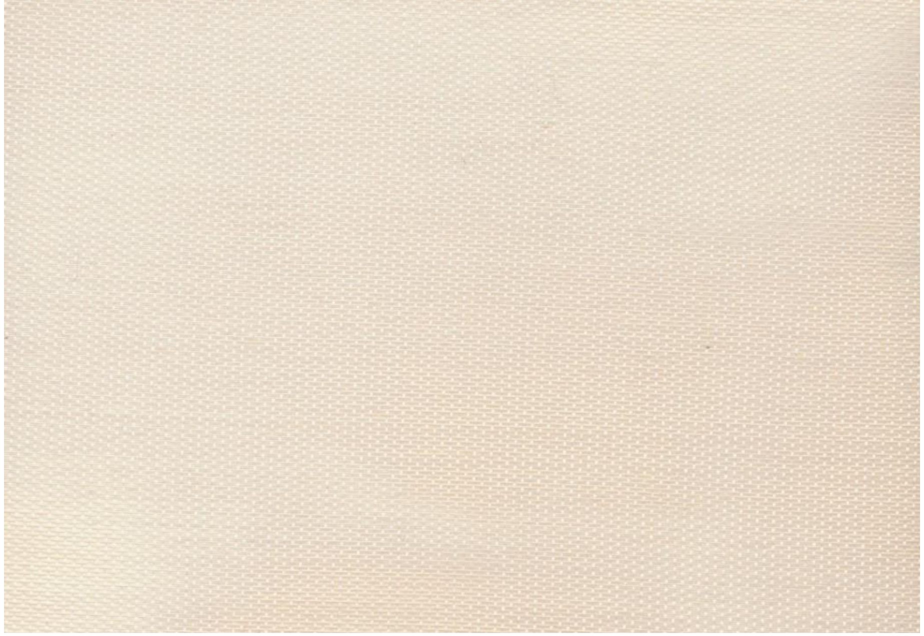

Mallastextiles Anti-Trip 55x30 lightgrey

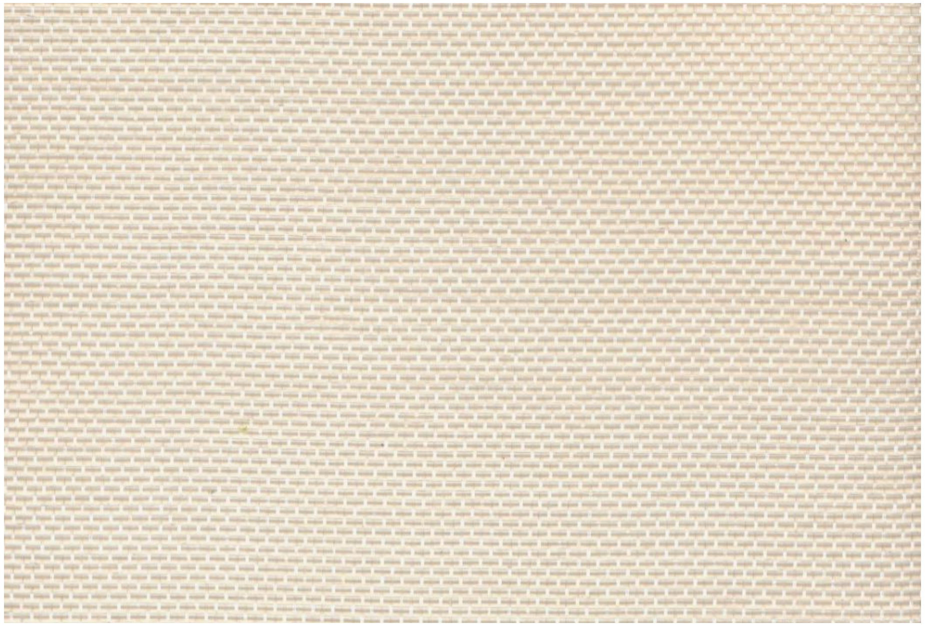

Mallastextiles Anti-Insectos 25x25 lightgrey

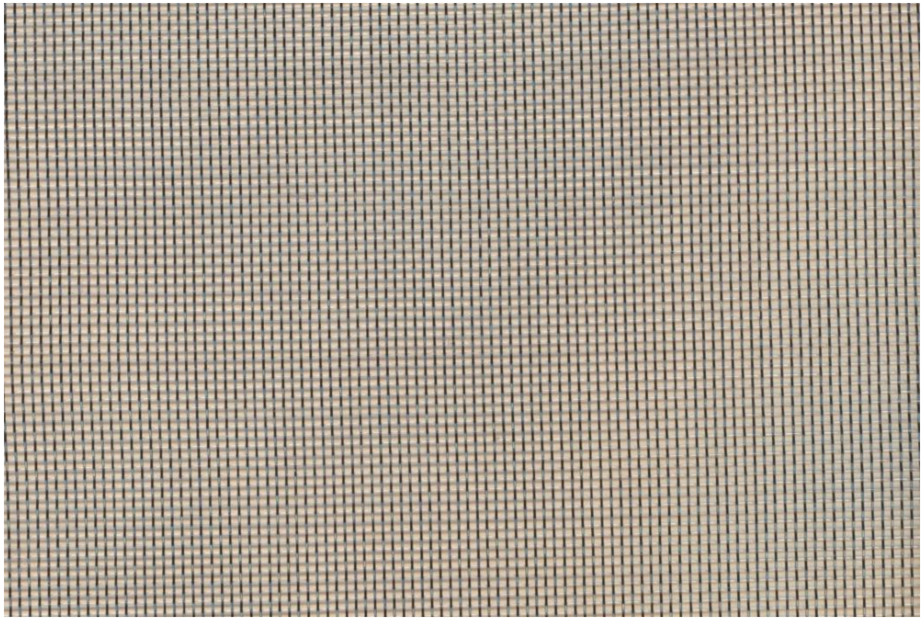

Mallastextiles Anti-Insectos 25x25 darkgrey

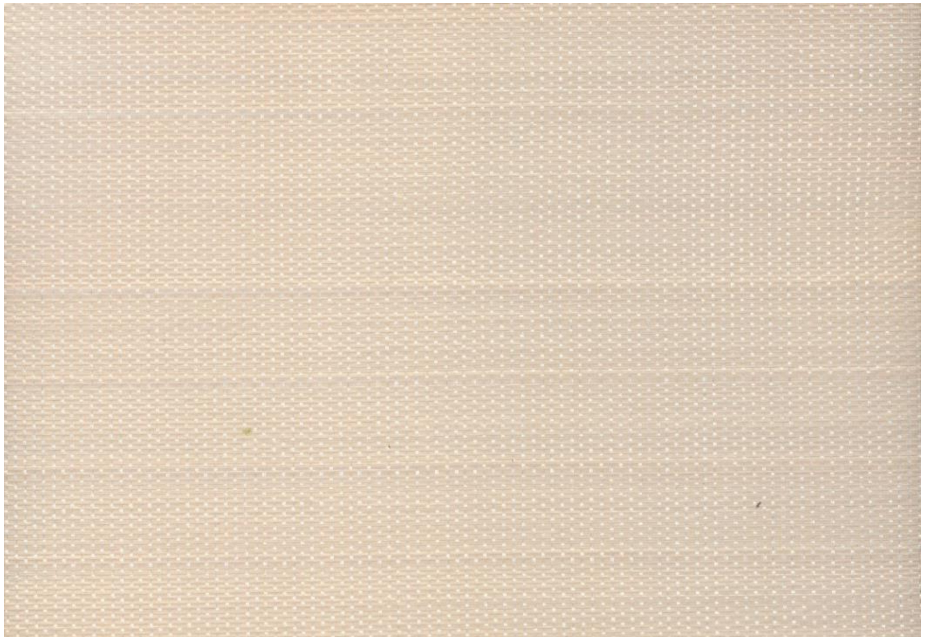

Mallastextiles Anti-Afidos 50x25 lightgrey

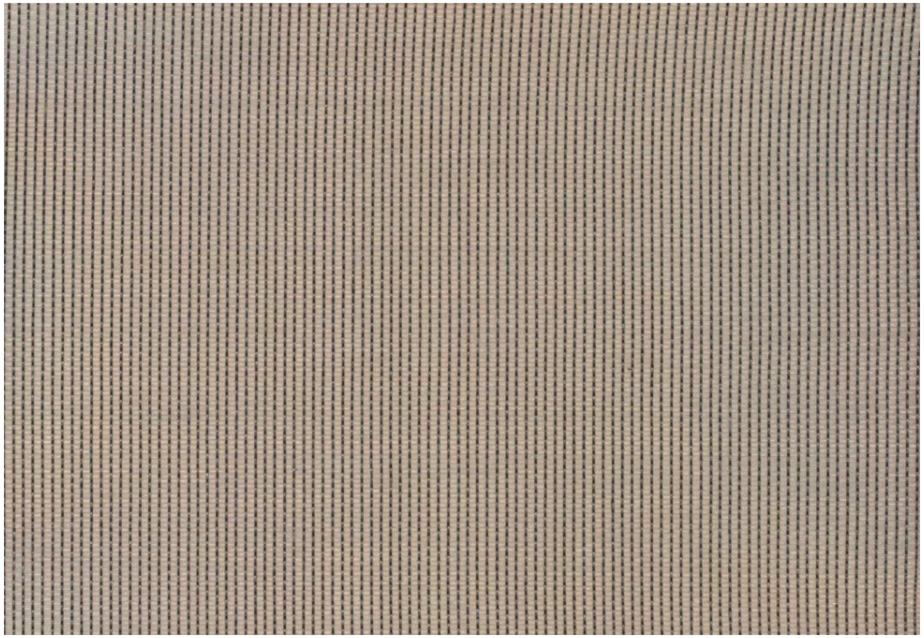

Mallastextiles Anti-Afidos 50x25 darkgrey

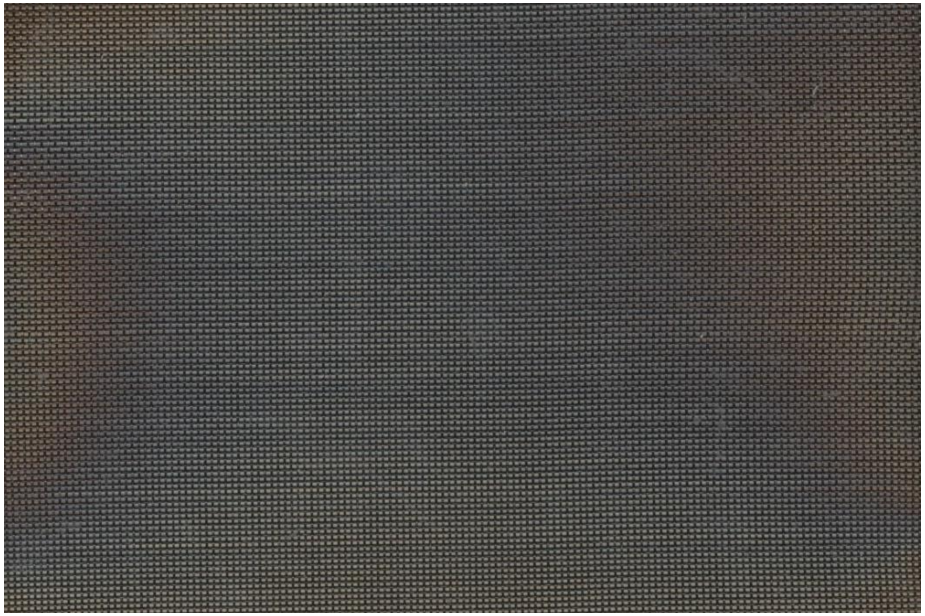

Mallastextiles Anti-Afidos 40x40 black

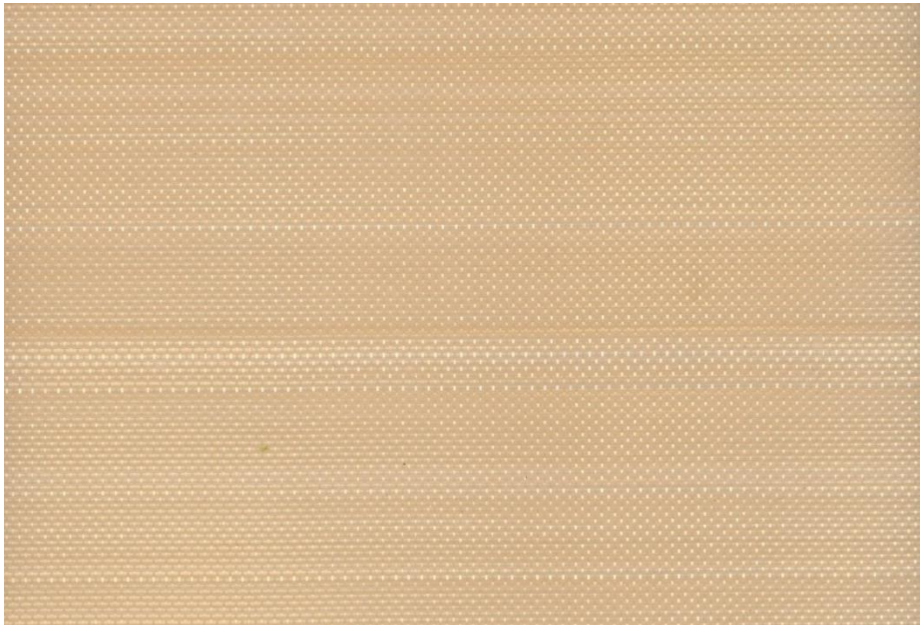

Mallastextiles Anti-Afidos 40x40 amber

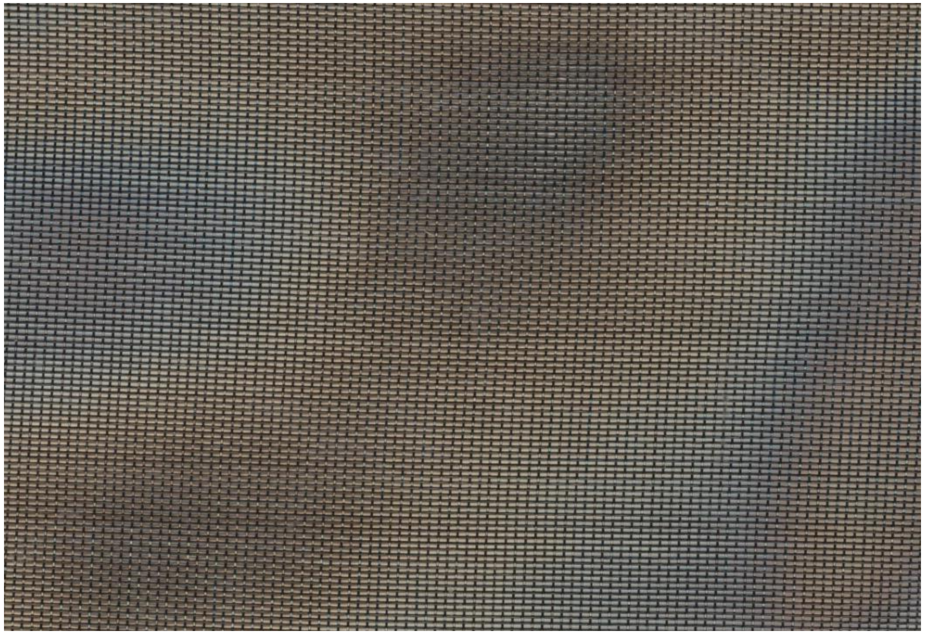

Mallastextiles Anti-Afidos 40x25 black

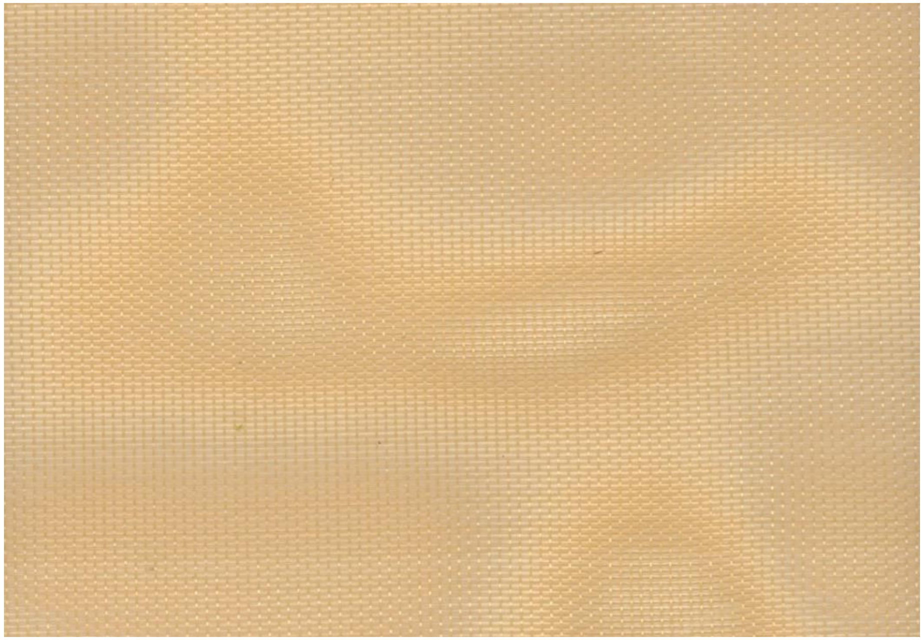

Mallastextiles Anti-Afidos 40x25 amber

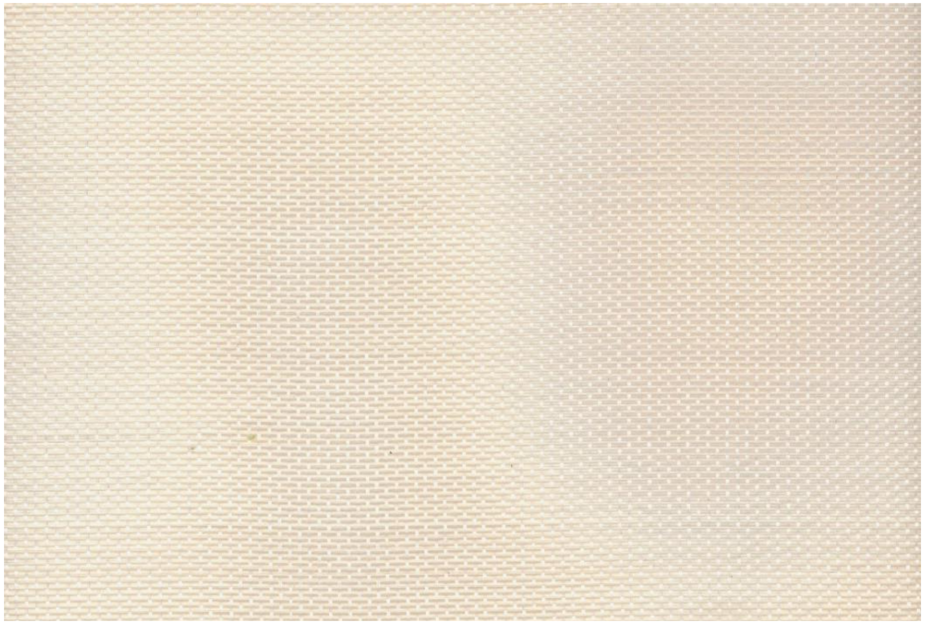

Mallastextiles Anti-Afidos 40x25 lightgrey

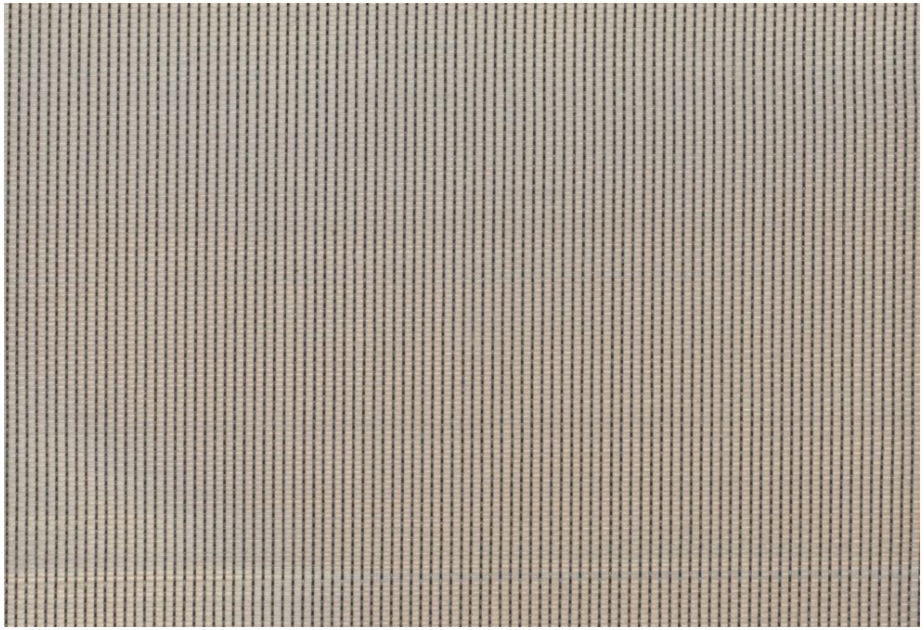

Mallastextiles Anti-Afidos 40x25 darkgrey

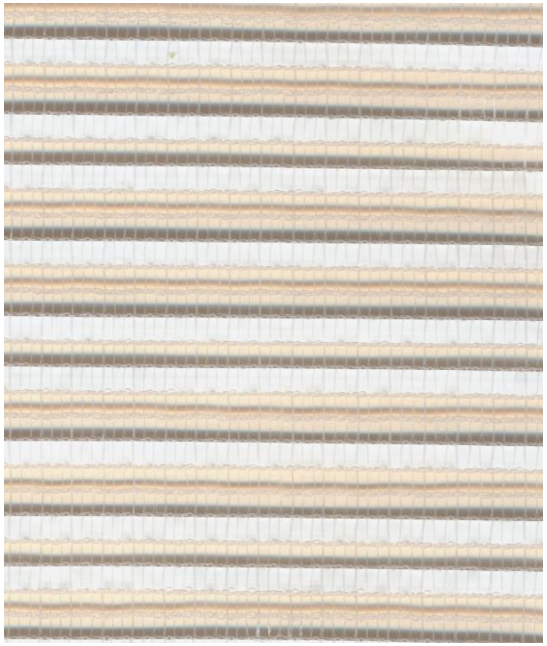

Svensson Harmony 3015

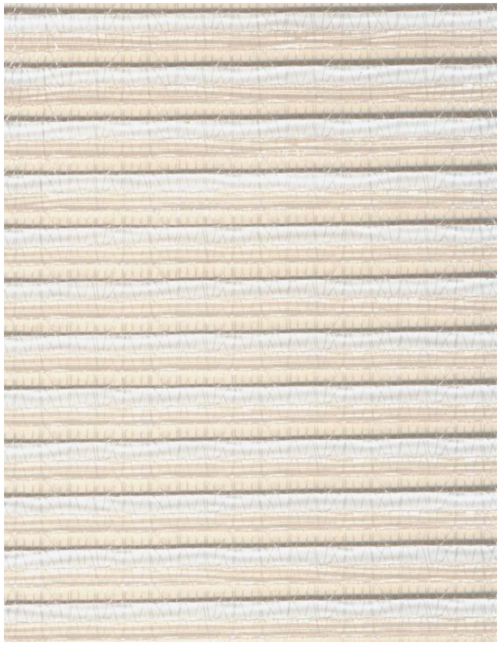

Svensson Harmony 3315

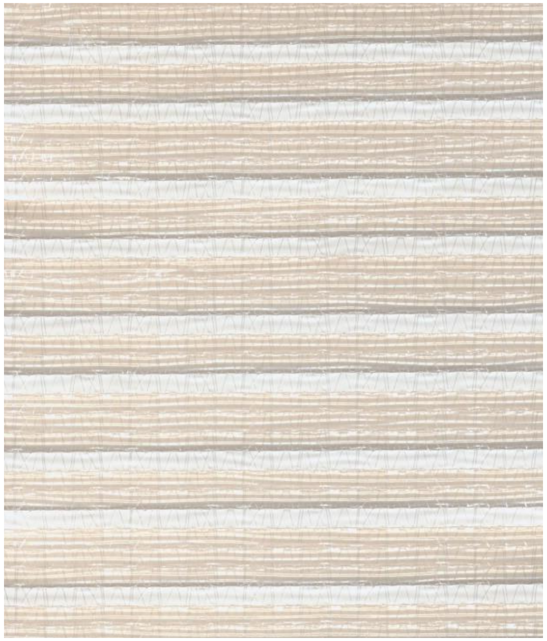

Svensson Harmony 3647

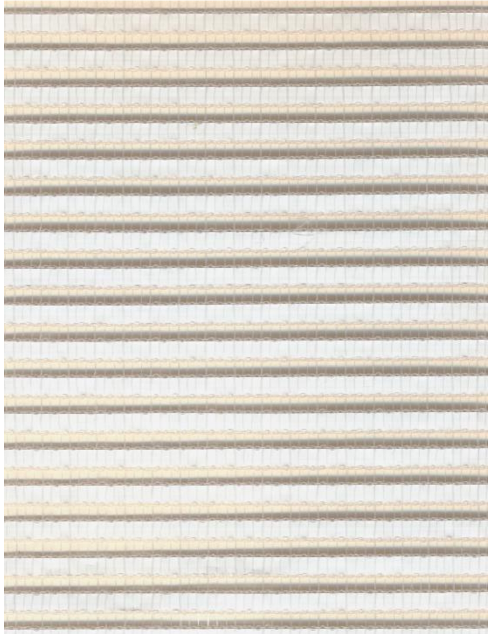

Svensson Harmony 3915

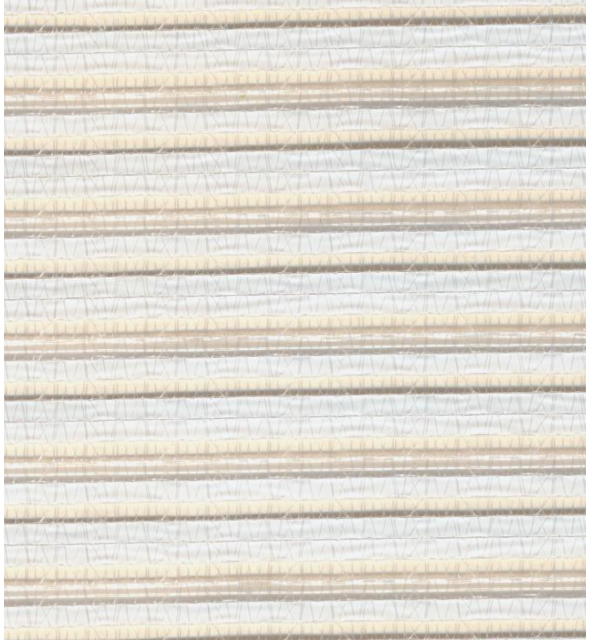

Svensson Harmony 4215

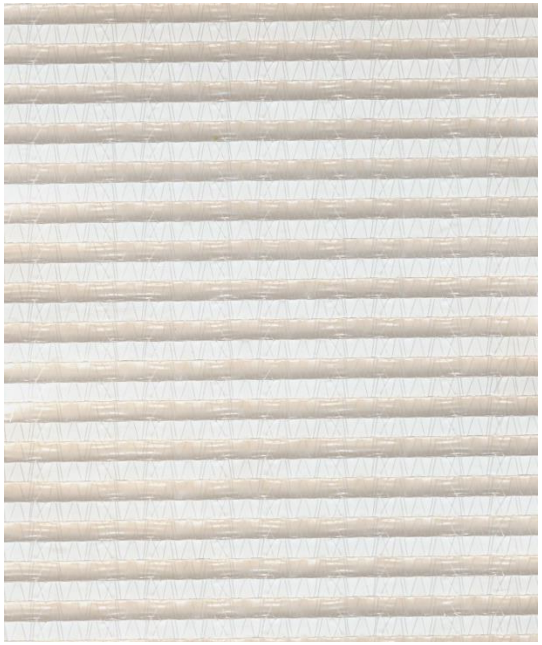

Svensson Harmony 4647

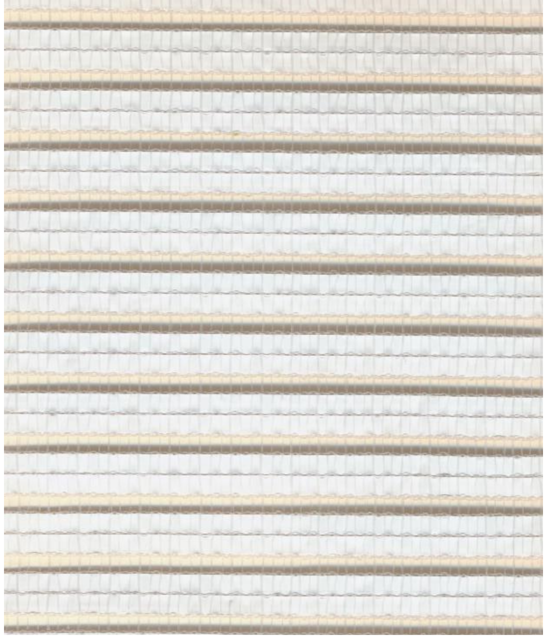

Svensson Harmony 5120

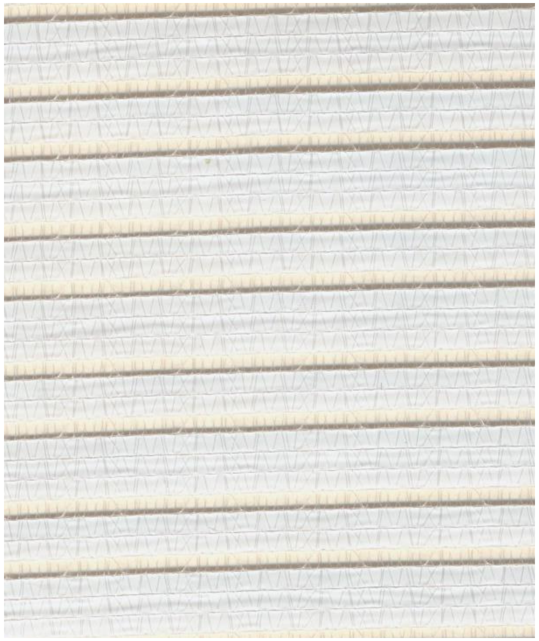

Svensson Harmony 5220

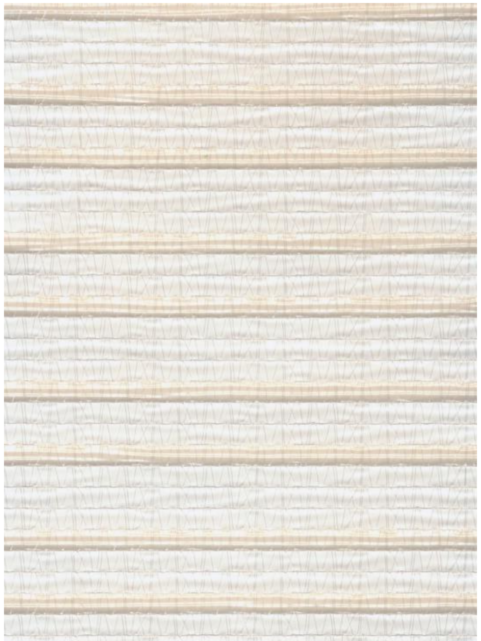

Svensson Harmony 5747

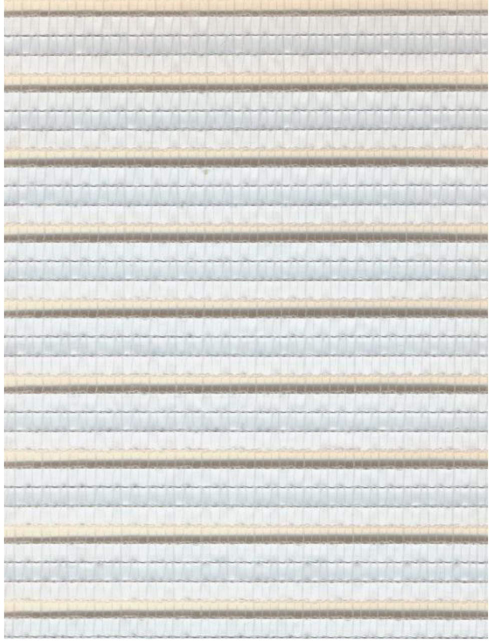

Svensson Harmony 6420

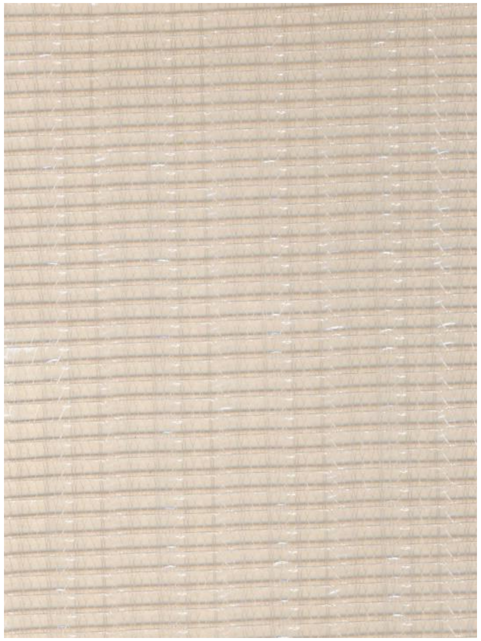

Svensson Luxous 1347

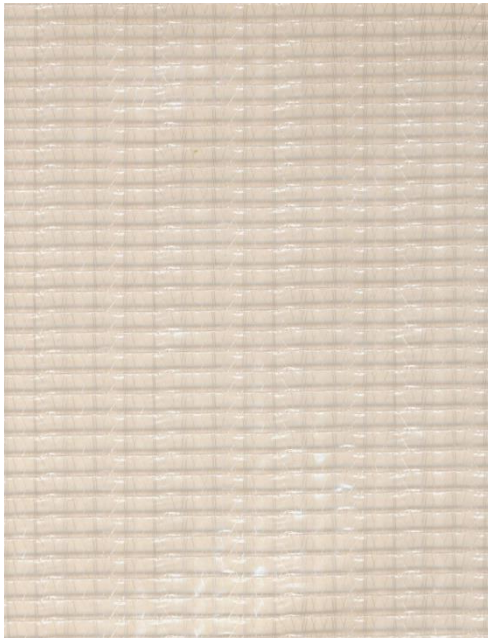

Svensson Luxous 1547

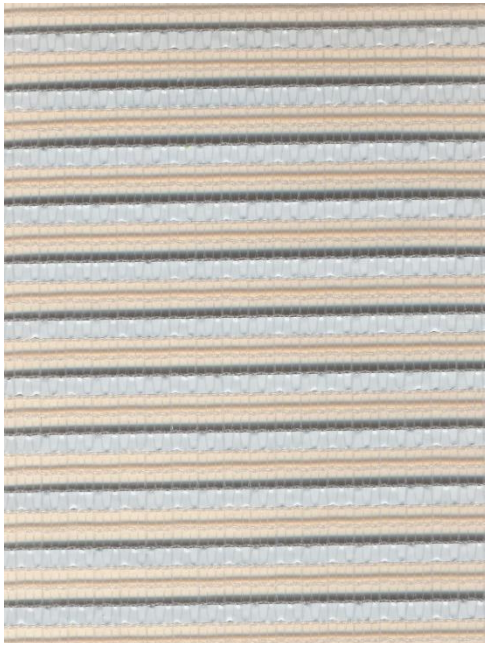

Svensson Solaro 3815

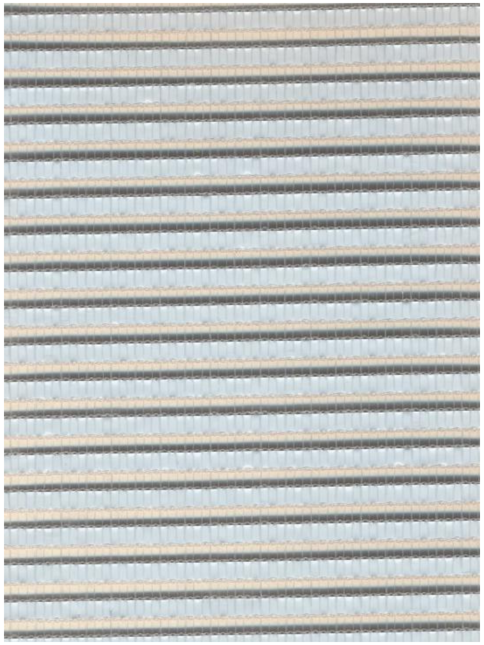

Svensson Solaro 5115

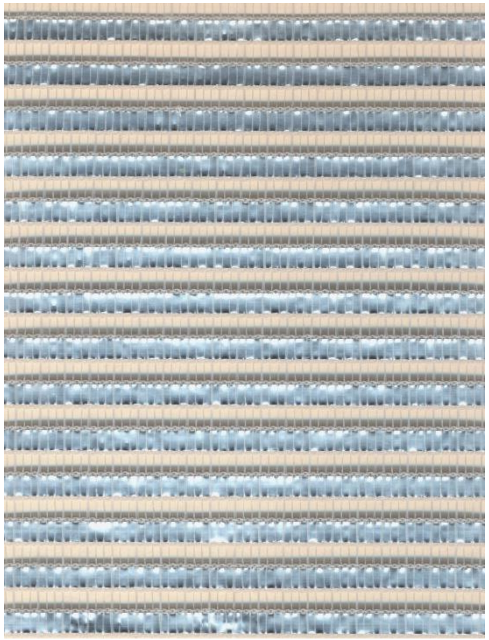

Svensson Solaro 5120

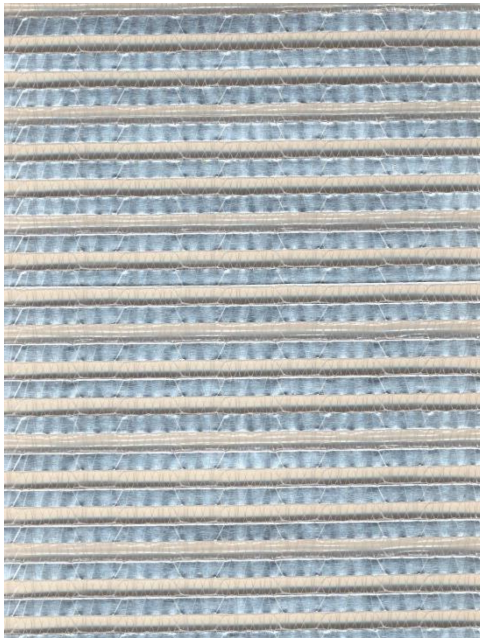

Svensson Solaro 5220

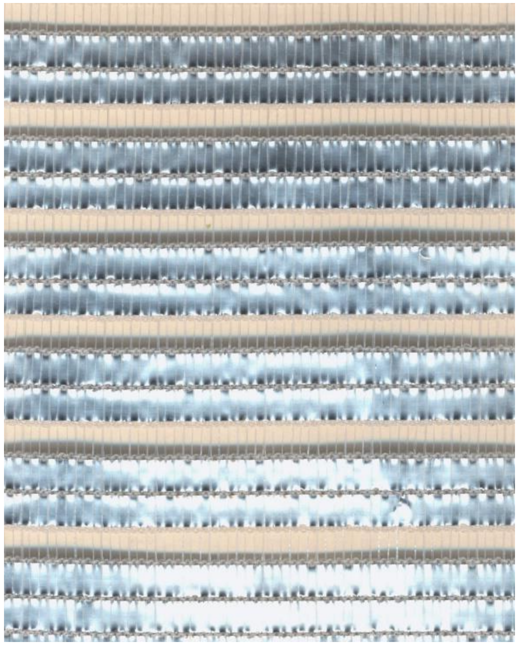
Svensson Solaro 6125

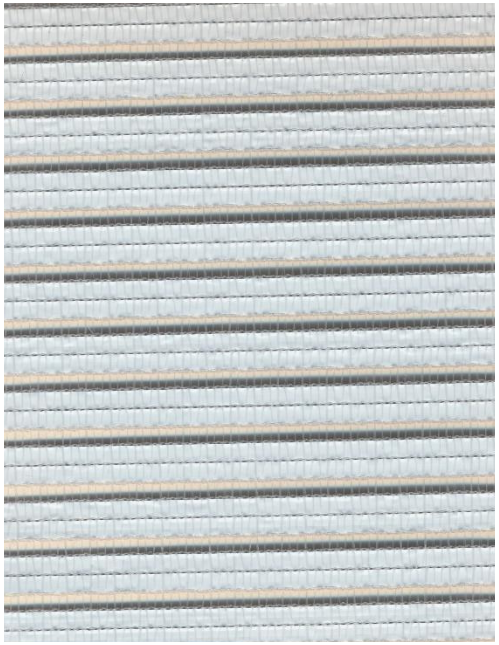
Svensson Solaro 6720

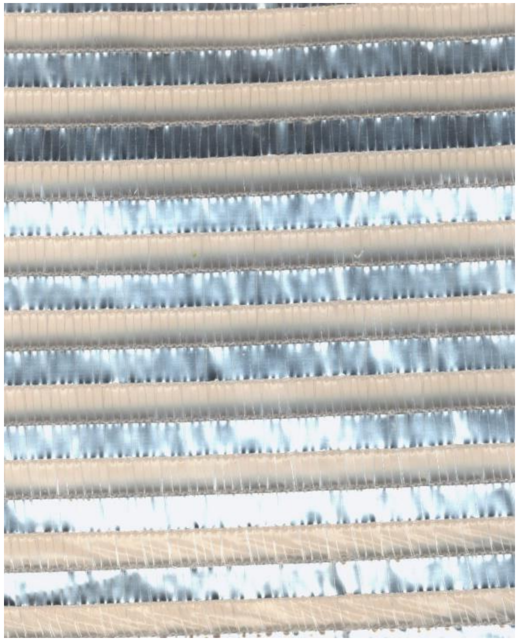
Svensson Tempa 5155

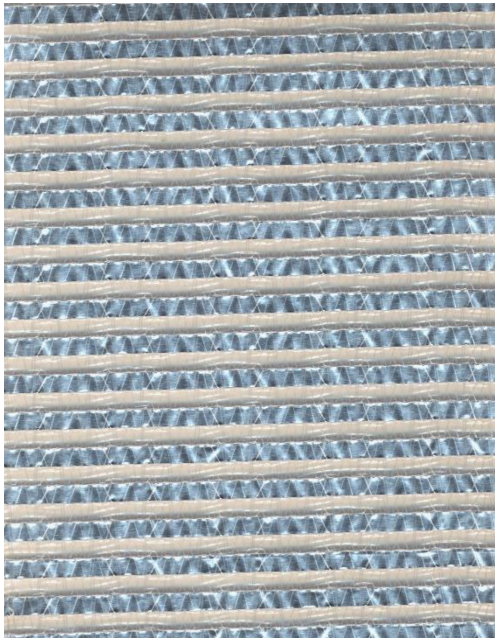
Svensson Tempa 5557

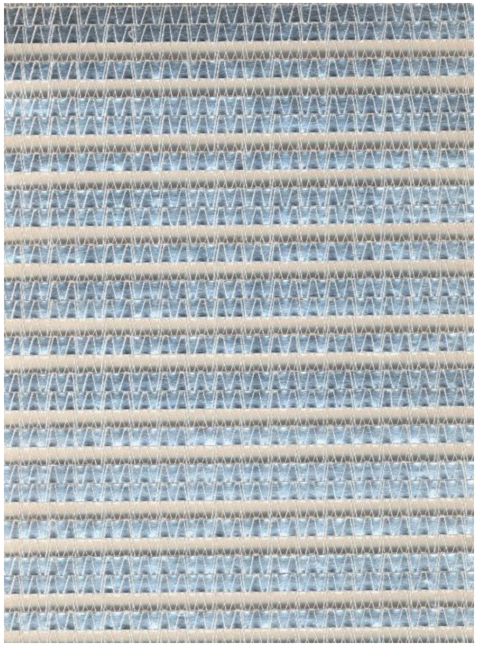

Svensson Tempa 6360

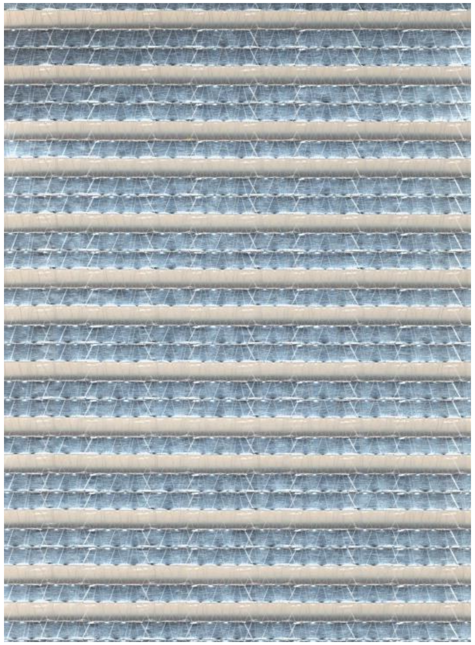

Svensson Tempa 6562

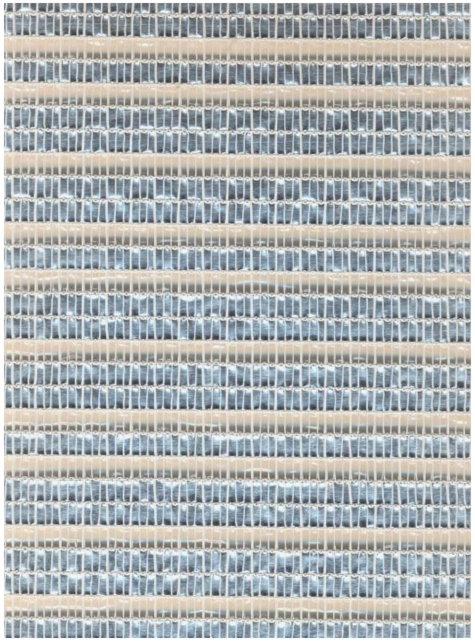

Svensson Tempa 6960

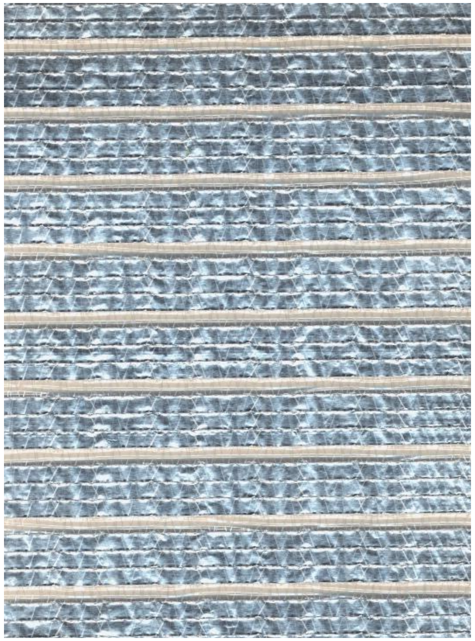

Svensson Tempa 7567

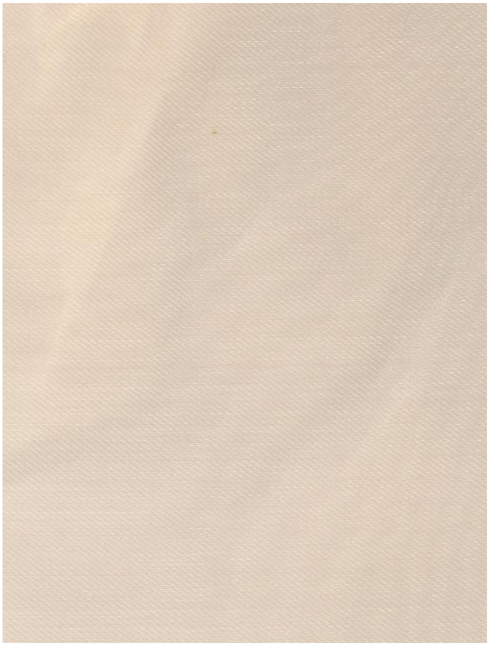

Svensson Insect 1515

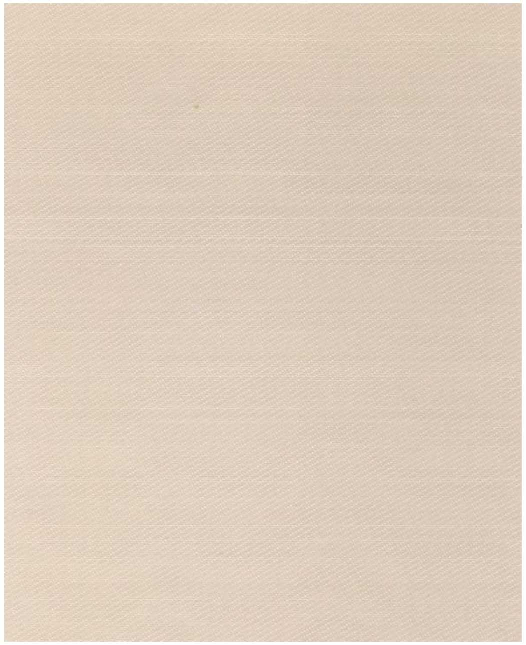

Svensson Insect 1535

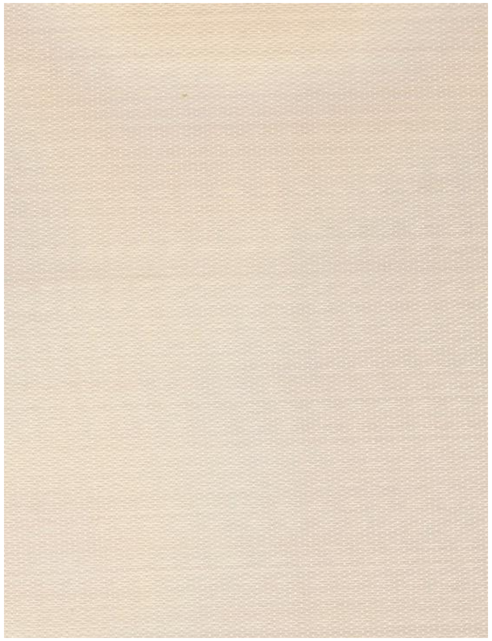

Svensson Insect 2777

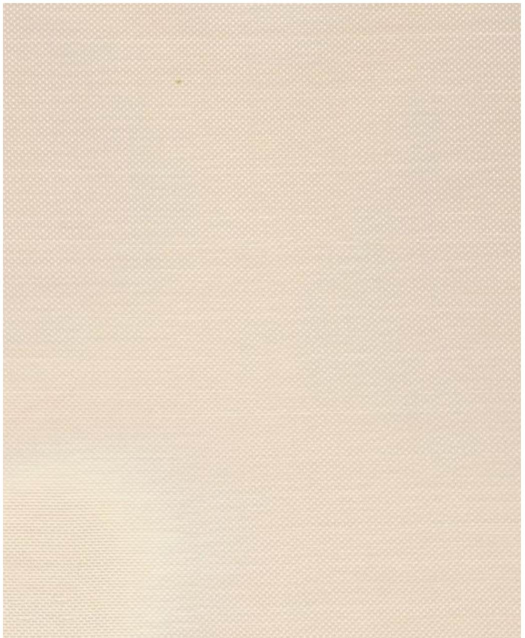

Svensson Insect 4045
